# Selection Criteria for De-Escalated Chemoradiotherapy for HPV-Related Oropharyngeal Cancer Based on Prognostic Biomarkers or Early Tumor Response to Therapy: A Narrative Review †

**DOI:** 10.3390/diagnostics16050674

**Published:** 2026-02-26

**Authors:** Avraham Eisbruch, M. P. Sreeram, Karthik Rao, Abbas Agaimy, Luiz P. Kowalski, Andrés Coca Pelaz, Anna Luíza Damaceno Araújo, Orlando Guntinas-Lichius, Juan P. Rodrigo, Fernando Lopez, Sandra Nuyts, Nabil F. Saba, Arlene Forastiere, Carol R. Bradford, Alfio Ferlito

**Affiliations:** 1 Department of Radiation Oncology, University of Michigan, Ann Arbor, MI 48109, USA; 2 Department of Head and Neck Oncology, Sri Shankara Cancer Foundation, Bangalore 560 004, India; sreeram.mp@sschrc.org (M.P.S.); karthik.nag.rao@gmail.com (K.R.); 3 Institute of Pathology, Friedrich-Alexander-University Erlangen-Nuremberg, University Hospital, 91054 Erlangen, Germany; al_ijaimy@yahoo.com; 4 Department of Head and Neck Surgery, University of São Paulo Medical School and AC Camargo Cancer Center, São Paulo 05403-000, Brazil; lp_kowalski@uol.com.br; 5 Department of Otolaryngology, Hospital Universitario Central de Asturias, University of Oviedo, ISPA, IUOPA, CIBERONC, 33011 Oviedo, Spain; acocapelaz@yahoo.es (A.C.P.); jprodrigo@uniovi.es (J.P.R.); fernandolopezphd@gmail.com (F.L.); 6 Head and Neck Surgery Department and LIM 28, University of São Paulo Medical School and Hospital Israelita Albert Einstein, São Paulo 05508-020, Brazil; anna_luizaf5ph@hotmail.com; 7 ENT Department, Jena University Hospital, 07747 Jena, Germany; orlando.guntinas@med.uni-jena.de; 8 Department of Radiation Oncology, Leuven Cancer Institute, UZ Leuven, 3000 Leuven, Belgium; sandra.nuyts@uzleuven.be; 9 Department of Hematology and Medical Oncology, Winship Cancer Institute, Emory University, Atlanta, GA 30322, USA; nfsaba@emory.edu; 10 The Sidney Kimmel Comprehensive Cancer Center at Johns Hopkins, Baltimore, MD 21231, USA; af@jhmi.edu; 11 College of Medicine, The Ohio State University, Columbus, OH 43201, USA; carol.bradford@osumc.edu; 12 Coordinator of the International Head and Neck Scientific Group, 35100 Padua, Italy; profalfioferlito@gmail.com

**Keywords:** HPV, oropharynx, chemo-radiotherapy, de-escalation, biomarkers

## Abstract

**Backgrounds**: Single-arm studies evaluating reduced intensity (de-escalated) therapy for low-risk Human Papillomavirus-related oropharyngeal cancer (HPV+OPC) patients demonstrated high cure rates and reduced toxicity compared with historical results of standard of care (SOC). However, randomized studies demonstrated that the outcomes of de-escalated therapies were inferior to standard therapy, suggesting that a minority of patients may not benefit from de-escalation. **Objectives**: to review strategies and prognostic biomarkers before or early during therapy to identify low-risk HPV+OPC patients who may require SOC and who should be excluded from de-escalation trials to avoid compromising outcomes. **Methods**: A comprehensive narrative literature review between January 2000 and August 2025 was performed to identify prognostic biomarkers in HPV+OPC, as well as studies reporting early-response indicators with prognostic potential in clinically defined good-prognosis HPV+OPC treated with chemo-irradiation. Preclinical studies were excluded unless their findings had implications for clinical outcomes. Data were synthesized qualitatively in this narrative report due to the substantial heterogeneity of the clinical and methodological aspects of the reviewed studies. The risk of bias in non-randomized studies was assessed using the Newcastle–Ottawa Scale (NOS) for cohort studies. **Results**: Multiple candidate prognostic biomarkers were identified, including molecular, histopathological, imaging, and clinical factors. Almost all studies were retrospective, included small cohorts and lacked internal or external validation, and had poor NOS scores, mostly due to lack of sufficient follow-up and lack of information about loss to follow-up, thereby precluding most biomarkers from current clinical utilization. Response-based selection based on induction chemotherapy is effective but limited by its added toxicity. Early tumor responses assessed by hypoxia, metabolic imaging, and circulating HPV DNA kinetics show encouraging preliminary results that need to be validated. **Conclusions**: Current evidence indicates major methodological limitations in most studies of prognostic biomarkers in clinically defined good-prognosis HPV+OPC. Early tumor response-based selection strategies are promising and warrant comparison with SOC in multi-center randomized trials.

## 1. Introduction

The rates of Human Papillomavirus (HPV)-related oropharyngeal cancer (HPV+OPC) cases have risen in developed countries in recent decades, reaching 71% of the OPC cases in the US, with 85–96% of these caused by HPV-16 infection [1]. Compared with HPV- unrelated OPC patients, whose cancers arise in the background of heavy smoking and alcohol consumption history, HPV+OPC patients are characterized by younger age, no or minimal tobacco and alcohol consumption history, fewer comorbidities, and better prognosis [2,3]. Clinically defined good-prognosis HPV+OPC are those with low stages and no or minimal smoking history [2]. Considering the high cure rates, relatively young age, and lack of comorbidities in good-prognosis HPV+OPC patients, efforts to mitigate sequelae while preserving the high cure rate of therapy have become an important goal of clinical research in this population. The prevailing “de-escalation” hypothesis is that reducing the intensity of therapy will achieve these goals.

Recent strategies employed in phase II studies of de-escalation in the definitive therapy of HPV+OPC used reduced radiotherapy (RT) doses to the primary tumors [4,5,6,7], eliminating concurrent cisplatin in all (ClinicalTrials.gov ID NCT06902623, [8]) or in a sub-set of patients [7], replacing cisplatin with cetuximab [9], reducing the extent of the elective targets irradiated prophylactically [10,11,12] and reducing both the extent and the doses to of these elective targets [13,14,15].

Most of these non-controlled phase II studies reported high tumor response rates, progression-free survival (PFS) and overall survival (OS), with toxicity levels considered beneficial compared with those expected following SOC chemo-radiotherapy (CRT). However, subsequent phase II–III randomized studies comparing SOC CRT to various de-escalation strategies in good-prognosis HPV+OPC have failed to demonstrate the non-inferiority of the de-escalated regimens. These randomized studies include comparing RT with concurrent cisplatin to RT concurrent with cetuximab [16,17,18,19], the delivery of RT alone [7], the delivery of reduced-dose RT concurrent with cisplatin, and the delivery of reduced-dose RT concurrent with Nivolumab [20]. In addition to these randomized studies, a recent meta-analysis reported inferior OS with treatment de-escalation in HPV+OPC compared with SOC [21]. These randomized studies suggest that a minority of the clinically defined good-prognosis HPV+OPC patients, approximately 10–15%, require SOC and should not undergo de-escalated therapy. It is therefore important to identify these patients and exclude them from de-escalation trials.

The realization that an early clinical stage and low smoking history as defining criteria for good-prognosis HPV+OPC patients may not be sufficient criteria for selecting patients for de-escalated therapy trials has led to recent efforts to improve patient selection. These efforts include utilizing empiric tumor response to chemotherapy or early response of tumor metabolic or genomic parameters and suggestions to explore biomarkers known to be prognostic in HPV+OPC and implement them in the selection process [22,23,24,25]. If such prognostic procedures or biomarkers are reliable, they will facilitate the exclusion of patients who likely require SOC from de-escalated trials and improve the outcomes of the entire HPV+OPC cohort. Furthermore, if a robust method exists to identify patients who may benefit from de-escalated therapy, despite lacking one or more clinical good-prognosis factors (e.g., smokers), it will allow for expansion of the pool of patients eligible for such therapy.

The objectives of this review are to assess whether the current published literature provides reliable methods to adequately select HPV+OPC patients for de-escalation studies. This review expands and clarifies the potential role of each of the strategies aiming to improve patient selection. These objectives are presented in the following themes: 1. patient selection based on early response to therapy, 2. clinical prognostic factors beyond smoking and early stage, 3. immune-related prognostic biomarkers, 4. molecular and genomic-related prognostic biomarkers, and 5. proteomic and metabolomic biomarkers.

## 2. Methods

A search of PubMed, MEDLINE, and SCOPUS databases was performed to identify molecular, histologic, radiologic, and clinical prognostic biomarkers in clinically defined good-prognosis HPV+OPC. The search covered peer-reviewed publications from January 2000 to August 2025 using the following keywords and Boolean operators: HPV, oropharynx, prognosis, chemoradiotherapy, de-escalation, p16, and biomarkers. Studies were included if they (1) specified the methodology used to confirm HPV positivity; studies that included p16 as the sole surrogate for HPV were retained, but interpretation prioritized those confirming transcriptionally active HPV through dual testing, (2) reported detailed tumor-related outcomes including hazard ratios or survival metrics, and (3) analyzed subgroups of HPV+OPC separately when mixed with HPV-unrelated populations. Preclinical or translational studies were included only if they provided findings with implications for clinical outcomes. When multiple reports from the same trial were available, the publication with the longest follow-up was used. The focus of the study centered on strategies associated with definitive chemo-radiotherapy as the primary therapy. The selection and reviews of the studies were made by the first author (AE). Data were synthesized qualitatively in this narrative report due to the substantial heterogeneity of the clinical and methodological aspects of the reviewed studies. The risk of bias in the non-randomized studies was assessed using the Newcastle–Ottawa Scale for cohort studies (NOS) [26].

For clarity, studies were grouped into these main categories: empirical approaches based on tumor response during therapy and approaches based on pre-therapy clinical, molecular, histological, and immunological prognostic biomarkers.

## 3. Results

A summary of the results is provided in Table 1.

### 3.1. Verification of HPV Status in p16+ OPC

The HPV oncogenic protein E7 causes inactivation of the retinoblastoma protein (RB), leading to p16 upregulation, and p16 overexpression is therefore a surrogate marker of HPV infection. However, in up to 15% of patients with p16+ OPC, HPV testing may be negative. Patients with discordant HPV and p16 positivity have a worse prognosis compared to those with both p16+ and proven HPV+ [107]. Recent de-escalation clinical trials, such as NRG HN002 and NRG HN005, determined risk stratification and eligibility based on p16 immunohistochemistry positivity alone, potentially harming recruited patients with p16-positive tumors that were not actually HPV driven. Dual testing with p16 immunohistochemistry and an HPV DNA or RNA test by Polymerase Chain Reaction (PCR) or in situ hybridization (ISH) should therefore be implemented as standard in trials that assess de-escalation strategies [27]. Additionally, testing for HPV E6 and E7 messenger RNA by ISH or PCR is considered by some authors as the gold standard for detecting transcriptionally active HPV virus [108]. However, these tests are not widely available clinically.

### 3.2. Empirical Approach: Studies Using Patient Selection for De-Escalation Based on Early Response to Therapy

#### 3.2.1. Patient Selection Based on the Response to Induction Chemotherapy

The selection of patients most likely to benefit from de-escalation has been made empirically by choosing those whose cancers demonstrated complete or significant partial responses following induction chemotherapy. This approach is based on early experience in advanced HNC, demonstrating that patients who had substantial tumor regression following induction enjoyed better prognosis compared with lesser responders [109]. This strategy was first incorporated in E1308, a phase II de-escalation study [28]. Patients with p16+ OPC received three cycles of induction chemotherapy followed by RT concurrent with cetuximab. Seventy percent achieved CR in the primary tumor and received a reduced dose—54 Gy—while those who did not achieve CR received a standard 70 Gy dose, resulting in a favorable disease control rate and functional outcomes in patients who received the lower dose (80% 2-year PFS). Subsequent adaptive studies from several institutions confirmed that responders treated with reduced dose and/or reduced irradiated target volumes maintained high disease control rates, including ≥95% DFS in some cases [4,28,30,110]. However, the delivery of induction chemotherapy was associated with added acute toxicity, competing with the potential benefits of the reduced definitive therapy intensity. This is of concern considering that induction chemotherapy does not confer any survival benefit to OPC patients [31,111].

#### 3.2.2. Patient Selection Based on Tumor Hypoxia

Pre-treatment high hypoxia tumor levels, assessed by tumor hypoxia-inducible factors (HIFs) in biopsies or in hypoxia-specific imaging such as 18-fluoromisonidazole PET/CT (^18^FMISO PET/CT), are adverse prognostic markers in most cancers. An even stronger adverse prognostic effect of driving hypoxia-mediated therapy resistance in HNC is residual hypoxia during therapy [112,113,114,115]. This effect is also observed in HPV+OPC [116,117].

The Memorial Sloan Kettering Cancer Center (MSKCC) group was the first to use these findings as a basis for de-escalation phase II studies [32]. Good-prognosis HPV+OPC patients underwent surgical removal of disease at their primary site but not of gross disease in the neck. A baseline ^18^F-MISO PET/CT was repeated 1–2 weeks into treatment, and patients with nonhypoxic tumors received 30 Gy over 3 weeks concurrent with chemotherapy, whereas those with residual hypoxic tumors received standard chemoradiotherapy of 70 Gy. Two-year progression-free survival (PFS) was 94% for the 30-Gy cohort, and a similar PFS was observed for the 70-Gy cohort. Acute grade 3–4 adverse events were much less common in the 30 Gy vs. the 70 Gy cohort, and late-grade 3–4 adverse events only occurred in the 70-Gy cohort. In an ongoing phase III MSKCC trial (NCT06563479), patients who are treated using the strategy implemented in the phase II study are randomized with patients receiving SOC regardless of hypoxia.

Using hypoxia and its early resolution as a biomarker to guide significant treatment intensity reduction has strong biological rationale, and the MSKCC phase III study will assess whether this strategy is non-inferior to SOC. However, broader implementation remains limited due to the lack of routine hypoxia-imaging capability in most centers.

#### 3.2.3. Patient Selection Based on Clinical Imaging of Tumor Metabolism

[^18^F] Fluorodeoxyglucose (FDG)-PET is a glucose analog that traces the early steps of glycolysis and has become integral and the standard of care in the staging and response assessment of HNC. Parameters of pre-treatment FDG-PET have been used to assess the prognosis of HPV+OPC, including Standard Uptake Value (SUV), Metabolic Tumor Volume (MTV), Total Lesion Glycolysis (TLG), and tumor-to-liver or tumor-to-blood ratios [118]. Several studies have demonstrated poorer prognosis in HPV+OPC patients based on these parameters and outperforming CT scans [119,120]. However, some studies have failed to observe the prognostic effect of baseline FDG-PET parameters in HPV+OPC patients while demonstrating their prognostic value only in HPV-unrelated patients [121,122]. There is strong evidence that the dynamics of FDG-PET during therapy have an additional, or superior, prognostic value compared to the pre-treatment parameters in HNC [123,124,125,126] and similar findings reported specifically for HPV+OPC [127,128,129,130].

The prognostic value of the relative change from baseline to the value obtained early during therapy in FDG-PET/CT parameters was utilized by investigators from the University of Michigan. In a phase II study of good-prognosis HPV+OPC, patients underwent repeat FDG-PET two weeks into CRT, and those with >50% reduction in MTV received a de-escalated dose of 54 Gy, while others completed 70 Gy; approximately half qualified for de-escalation, and outcomes showed low 2-year LRF rates with notably reduced swallowing toxicity in the de-escalated group [33,34], A similar approach is being tested at Duke University, where intra-treatment FDG-PET/CT at two weeks is being used to identify early responders for dose reduction. The wide availability of FDG-PET should support a multi-institutional randomized study comparing this strategy with SOC.

#### 3.2.4. Circulating Tumor DNA (ctDNA) and Their Response During Therapy

ctDNA refers to fragments of DNA released into circulation following tumor apoptosis or necrosis. Next-generation sequencing (NGS)-based techniques that capture, amplify, and quantify the amount of ctHPV-DNA in plasma samples, combined with digital PCR, allow for the detection and quantitation of trace amounts of ctDNA [131]. In early HPV+OPC, ctHPV DNA baseline plasma levels are detected at a high rate [132] and represent a non-invasive approach to assess tumor burden and its dynamics during therapy. A correlation between clinical tumor burden and ctHPV DNA has been reported in several studies [133,134,135,136]. However, Chera et al. reported that while low baseline levels of HPV16 ctDNA (≤200 copies/mL) were associated with low tumor burden, they were also associated with worse prognosis, because they were derived from cells with a lower tumor HPV copy number and higher HPV integration, which are adverse prognostic factors [35,36].

The dynamics of ctHPV DNA during therapy may have a better prognostic value compared to those of the pre-treatment levels. In the study by Chera et al., a ctHPV16DNA favorable clearance profile was defined as having high baseline copy numbers and >95% clearance of ctHPV16DNA by the fourth week of CRT. None of the 19/67 evaluable patients with favorable ctHPV16DNA clearance recurred after CRT compared with the 35% recurrence rate in patients with unfavorable ctHPV16DNA clearance [35]. Similar results regarding the value of the kinetics of ctHPV-DNA were reported by others [37]. Issues related to differing results in these studies include different methods used for the detection of ctHPV, having different sensitivities and specificities. While the prognostic value of ctDNA clearance is clear, the lack of standardized assays and defined thresholds remains a significant hurdle to its widespread implementation [137]. Several ongoing phase Il trials are currently investigating ctDNA kinetics during therapy as a biomarker for treatment de-escalation in HPV+OPC: NCT05307939, NCT04900623, NCT05541016. The results of these studies will provide clearer evidence for the utility of the dynamics of cfHPV DNA for selecting patients for de-escalation trials.

In addition to quantitating ctDNA, they have the potential to serve as a “liquid biopsy” to assess the mutational and epigenetic profile of HPV+OPC tumors. However, the likelihood of mutational variant detection in HPV+OPC using PCR is low [67,68]. Newer technology may improve detection; the recent use of HPV whole genome next-generation sequencing (NGS) liquid biopsy has enabled the detection of prognostic features including high-risk HPV16 SNPs, viral integration events, and PIK3CA mutations [69]. Similarly, circulating tumor cells (CTCs) have been reported to be prognostic in HNC; however, they have a very low abundance in HPV+OPC [70]. Exosomes are membranous extracellular vesicles released by tumor cells carrying a variety of bioactive substances, including proteins, RNA, DNA, miRNA, lipids, and metabolites, providing potential prognostic biomarkers in HPV+OPC [138]. None of these exosome-carried biomarkers have been validated, and some were abundant in HPV+OPC patients’ blood but were absent in tumor cells, suggesting probable secretion by non-cancer cells like immune or stromal cells [139].

In conclusion, while studies evaluating the kinetics of ctHPV DNA are ongoing and await validation, prognostic biomarkers obtained from “liquid biopsies” are currently unlikely to be of value in selecting HPV+OPC patients; however, the adoption of newer technologies like NGS and digital droplet PCR (ddPCR) [140] is promising.

### 3.3. Clinical, Radiological, and Histological Biomarkers

#### 3.3.1. Imaging-Based Extranodal Extension (iENE)

While matted nodes are a well-known adverse prognostic factor used as an exclusion criterion in recent de-escalation studies [43], subtle iENE is not regularly considered. A meta-analysis including eighteen studies and 3603 HPV+OPC patients, about a third of whom were iENE+, found that the presence of iENE was associated with decreased OS and increased distant recurrence [44]. Data from Princess Margaret Hospital suggested that iENE is an adverse prognostic factor that may justify upstaging N1 to a higher N classification in AJCC v8 [45]. This proposal was tested in an international multi-institutional review of 2053 HPV+OPC patients, where neuroradiologists reviewed CT and MRI images retrospectively [46]. They identified 1898 patients with nodal disease, 37.4% of whom had iENE; an MVA model that included all radiologic nodal features showed that iENE was the strongest prognostic factor for OS. iENE-positive N1 disease was therefore reclassified in this proposal as N2 disease and improved the separation of OS in stage I vs. stage II compared with the current TNM staging. These findings were validated in an independent HPV+OPC cohort.

The prognostic power of iENE in HPV+OPC in real-world practice was determined in a retrospective cohort of 821 consecutive subjects with p16 + OPC from 13 multinational tertiary care hospitals in nine countries [47]. Diagnosis of iENE was made by local radiologists in routine clinical practice. On univariate analysis, cases with iENE had worse DFS and OS compared to iENE negative, but on multivariant analysis (MVA), iENE positivity was not a statistically significant predictor of either. When pathologically based ENE was compared to iENE in patients who had nodal dissections, significantly increased sensitivity (84.6%) and specificity (94.5%) were observed if patients had both CT and MRI compared with only CT (sensitivity 47%, specificity 78%) or MRI (sensitivity 18%, specificity 96%) alone. Specialist neuroradiologists showed better specificity and similar sensitivity compared to nonspecialists.

Radiomics and machine learning may further standardize iENE assessment. Retrospective evaluation of deep learning algorithm performance using pre-treatment CT scans of patients accrued to the postoperative dose de-escalation E3311 study was compared to the surgical pathology reports [141]. This study found high variability in specificity and sensitivity among radiologists, with poor inter-reader agreement. The algorithm showed improved performance compared with that of the radiologists.

Improved methods to identify patients with iENE are required, including using both CT and MRI and relying on expert neuroradiologists [142]. Excluding these patients is likely to improve the outcomes of de-escalation studies.

#### 3.3.2. Radiomics

Radiomics represents a quantitative image analysis using features that are extracted algorithmically to correlate with underlying tumor biology, gaining significant information that could not be acquired through human analysis alone [143]. The common texture features determined by radiomics capture tissue and lesion properties such as shape and heterogeneity. Radiomic features are strongly correlated with heterogeneity indices at the cellular level, which have a prognostic impact [144], as well as a link to gene expression and mutation levels [145]. In OPC-related radiomics studies, most investigators examined its utility to differentiate between HPV+ and HPV-patients. Some reported satisfactory results [146], while others reported no significant association between radiomic signature and HPV status [147]. Several studies found that the combination of radiomics, HPV status, and clinical factors improved the prognostic prediction of OPC patients compared to HPV status and clinical factors alone, suggesting that radiomics may be a valuable tool in selecting patients for de-escalation [119,148,149,150,151], However, other studies could not verify the value of radiomics as a prognostic factor [152,153]. Moreover, a negative study has also been reported. Adding radiomic variables to a model that included HPV status and clinical factors actually worsened the predictive value of the clinical model alone [154].

Several weaknesses in the current radiomics literature may explain the contrasting outcomes, including different methods of segmentation, feature selection, or modeling, by various institutions [144]. Recent systematic reviews of radiomics in HPV+OPC concluded that the quality of the research methodologies varies and is a limiting factor for its broader clinical application [38,39].

#### 3.3.3. Histopathological Morphology

Morphological features of hematoxylin and eosin (H&E) histology staining have been reported to offer prognostic information in HPV+OPC. Lewis et al. [155] reported that anaplasia and multinucleation in a p16-positive cohort, in which half the patients had those features, were associated with poor outcomes. A machine learning approach to quantify tumor multinucleation from digitally scanned H&E-stained slides was used to optimize and validate a multinucleation index (MuNi) [156]. The MuNI was prognostic for DFS and OS in MVP, including in stage I patients. However, the prognostic value of anaplasia and multinuclearity in HPV +OPC has been challenged by other studies [42,157].

Image analysis using machine learning (ML) and Artificial Intelligence (AI) algorithms to quantify the spatial patterns of tumor infiltrating lymphocytes (TILs) and surrounding nucleated cancer cells on diagnostic H&E biopsy slides from HPV+OPC have been used by several groups reporting significant correlations between these patterns and outcomes [40,41,156,158]. In general, intra-tumor homogeneity for various measurements was found in favorable outcomes, while greater heterogeneity is unfavorable. However, in a study using automated image analysis of diagnostic H&E slides, none of the variables alone was predictive of the outcome [159]. In conclusion, while automated histology analysis shows promise for uncovering prognostically significant spatial relationships in the tumor microenvironment (TME), the current lack of standardized, validated algorithms prevents its clinical adoption.

### 3.4. Immune-Related Biomarkers

#### 3.4.1. Neutrophil/Lymphocyte Ratio

Pre-treatment hematological parameters, including total circulating lymphocyte count (TLC), circulating monocytes (TMC), circulating neutrophil count (TNC), and the neutrophil-lymphocyte ratio (NLR), have been examined as potential prognostic markers. A large recent study from Princess Margaret Hospital included data from 1151 HPV+OPC patients treated with RT or chemo-RT, and the prognostic impact of pre-treatment TLC, TNC, NLR, and TMC was assessed [48]. Pretreatment TLC was a good prognostic factor for HPV+OPC patients receiving CRT but not RT alone. NLR was significant for worse OS in the cohort receiving CRT, but not for patients receiving RT alone. The authors hypothesized that TLC may impact the ability to mount a cisplatin-potentiated immune response in the lymphatic-rich HPV+OPC via migration of lymphocytes to the TME. Similar findings were reported by others [49]. The conclusions from these data are that a low TLC is a poor prognostic factor for patients receiving radiotherapy alone but is attenuated by cisplatin chemotherapy. NLR, a marker of inflammation, has been reported in some studies to be an adverse prognostic factor in HPV+OPC [50,160]; however, conflicting data suggests its value in HNC but not in the HPV+OPC sub-population [161]. Limitations include the potential effects of comorbidities and advanced malignant disease on NLR, and large inter-individual variations in the blood components, such that decision-making based on a threshold of any single hematological parameter may not be feasible.

#### 3.4.2. Tumor Infiltrating Lymphocytes (TILs)

Studies of the immune components of the TME may stratify HPV+OPC populations and identify the minority of patients with a less favorable prognosis [51,162]. High TIL density in the primary tumor and stroma was found to be a statistically significant prognostic factor; however, the concordance between the pathologists assessing TIL density was moderate [163]. On the other hand, others reported that stromal, rather than tumor, TILs were linked to improved survival [164]. The Mayo Clinic group integrated H&E staining and immunohistochemistry (IHC) in HPV+OPC to identify immune-based patient subgroups based on the spatial distribution of TILs in the stroma or in the tumor [52]. They reported distinct immune subtypes that were significantly associated with PFS. Notably, the digital pathology and deep learning approach identified a substantially higher number of patients with a favorable prognosis than the manual approach. No validation of their results has been performed.

The University of Western Ontario group created a 3-gene immune score, UWO3, that classified HPV+OPC patients into immune-rich, mixed, or “immune desert”, and higher score was correlated with worse tumor outcomes [165]. Importantly, the UWO3 score was associated with higher odds of recurrence following aggressive treatment de-escalation. The extensive validation of this immune signature in multiple cohorts in that study, and the readily clinical availability of the immunohistochemical methods, are promising if future studies concur with these results.

Rapid alterations in immune cells during the early treatment in HPV+OPC patients receiving cetuximab were reported to predict eventual response, potentially aiding in adaptive therapy [166,167]. Whether such changes apply to patients receiving chemo-RT is not known.

#### 3.4.3. Immune vs. Keratinized TME

Efforts to segregate HPV+OPC based on immune signatures and molecular features beyond the abundance of TILS have been made Including segregation into immune and keratinization subtypes characteristics [53]. Immune-rich tumors (IMU) have more mesenchymal differentiation and more epithelial-to-mesenchymal (EMT) differentiation. The keratinization (KRT) subtype is uncommon in HPV+OPC, associated usually with smoking, demonstrates more HPV integration into the host genome and carries a significantly worse prognosis which may be related to smoking [54,168,169]. The association of KRT with heavy smoking history likely excludes this category from consideration as an independent factor.

#### 3.4.4. HPV Related Proteins and Their Antibodies

E6 and E7 are HPV virus-produced proteins that suppress the tumor regulators p53 and Rb. Other oncogenic functions of E6 and E7 include compromising cellular DNA repair, enhancing genomic instability, and increasing immune escape [55]. Contradictory prognostic effects of the presence or absence of serology against HPV proteins have been reported. Pre-treatment serum antibodies to HPV E6 and E7 antigens were associated with favorable prognosis in several studies [56,57,58]. In contrast, others reported that HPV serology is associated with worse prognosis [59,60,61].

While the main oncogenes of HPV are E6 and E7, E6 has isoform variations that may affect prognosis. A full-length variant (E6FL) is expressed in all HPV types, but it is also expressed in some HPV+ tumors in shorter spliced isoform known as E6*, whose presence confers worse prognosis and promotes cancer progression [62]. An explanation for the adverse effect of E6* on outcomes is that it may interfere with p53 inhibition by the full E6 isoform [170]. It is possible that the contradictory reports regarding the prognosis of HPV serology will be clarified once the isoforms of E6 and their antibodies will be fully considered in clinical studies. Thus far little has been reported.

#### 3.4.5. PD-L1 Levels

While PD-L1 status is associated with the response of HNC to immunotherapy, its expression in relation to the response and prognosis after surgery or chemo-RT is less clear. In a meta-analysis of the prognostic significance of PD-L1 expression in OPC patients following standard, non-immunotherapy treatment, Polesei et al. reported A total of 1522 OPSCC patients from 12 studies [63]. Different cut-off values for the Tumor Proportion Scores (TPS), defined as the percentage of PD-L1-positive tumor cells, were utilized in the different studies ranging from 1% to 50%. Only one of the studies used the Combined Positive Score (CPS) ≥ 1% to define a PD-L1 positive OPC. A difference in OS related to PD-L1 expression reached statistical significance only in HPV+OPC, where a 60% reduction in mortality in patients who expressed PD-L1 compared to those who did not was observed.

As PD-L1 gets up-regulated in response to tumor-infiltrating lymphocytes (TIL)-derived cytokines, the increase in PD-L1 expression in HPV+OPC might be related to a more inflamed tumor microenvironment with higher numbers of TILs. HPV+OPC tumors with both high TILs and high PD-L1 expression had especially favorable outcomes [64].

While the better prognosis of patients with PD-L1 TPS > 5% in the study of Solomon et al. is encouraging, the threshold of 5% that was found to be statistically significant leaves more than half the patients in the poor prognosis category in this study. A much larger study may validate whether a PD-L1 combined score of ≤1%, which confers poorer treatment response to patients receiving immunotherapy, is also relevant to patients receiving standard therapy.

### 3.5. Molecular Biomarkers

#### 3.5.1. Head and Neck Cancer Stem Cells

In 2007 Prince et al. first reported the existence of a subpopulation of head and neck cancer stem cells (HNCSC), expressing cell-surface protein CD44 [65]. Stem cell-like properties included self-renewal, tumorigenesis, and differentiation into non-stem cells, and their frequency in tumors are associated with high tumor grade and poor outcome [65]. Additional HNCSC markers have subsequently been identified such as CD133, CD98, and others [171]. Within HPV+OPC group, a high percentage of CD98+ tumor cells (>50%) was reported to be associated with significantly worse 5-year OS and PFS compared to patients with a lower percentage, but the percentage of CD44-positive cells did not correlate with outcomes [172]. In contrast, another study reported that HPV+OPC patients with CD44 absent/weak expression displayed significantly favorable 3-year DFS and OS [66]. Kim et al. developed and validated an expression signature of HNCSC biomarker genes whose mRNA expression levels correlated with at least one of the four cancer stem cell genes [173]. There were no significant differences in the 5-year OS rates between the high and low expression levels subgroups in the HPV+OPC patients, while a significant difference was observed among the HPV-negative OPC patients. They remarked that a strength of their study was using a 4-group HNCSC marker signature vs. a single marker used by other groups.

In summary, while qualitatively, high expression of HNCSC is an adverse prognostic marker in most studies of HPV+OPC, there are multiple unresolved issues regarding the thresholds defining high vs. low expression and regarding the use of single vs. multiple markers of HNCSC. In addition, the relatively large percentage of HPV+OPC patients harboring high expression of HNCSC markers, reported in the various studies, excludes them from being an isolated factor selecting high-risk, good-prognosis HPV+OPC patients.

#### 3.5.2. TP53

p53 is a tumor suppressor protein produced by the TP53 gene, preventing DNA damage repair and triggering apoptosis in damaged cancer cells. HPV E6 protein binds and degrades p53, causing reduced levels and abrogating its function. In a seminal study of 560 HNC patients enrolled in prospective ECOG and RTOG studies, the presence of TP53 mutation was significantly associated with decreased OS compared with wild type [174]. Following classification of the mutations into disruptive (mutations located inside the key DNA-binding domain L2–L3 region, or stop codons in any region), and nondisruptive (mutations outside the L2–L3 region), the decrease in survival was limited to the patients with disruptive mutations [174]. Several studies have corroborated these findings [175]. While disruptive TP53 mutations are common in non-HPV related OPC and are associated with poor outcomes, these mutations are rare or even not found in HPV+OPC [79,80].

Based on the data about the rarity of disruptive TP53 mutations in HPV+OPC, its status may not affect decisions about de-escalation therapy.

#### 3.5.3. EGFR Expression and Mutations

EGFR (Epidermal growth factor receptor) is a receptor tyrosine kinase overexpressed in many cancers; its activation leads to auto-phosphorylation triggering a downstream cascade stimulating cell division. There is an inverse correlation between EGFR gene copy number and protein expression vs. HPV copy number, and a significant correlation between the presence of phosphorylated EGFR, its activated form, and HPV-negative OPC [81]. In several studies EGFR expression was high in about a third of HPV+OPC patients, but in most studies high expression was associated with worse prognosis only in HPV-negative OPC [82,83,84], contrasting with other studies demonstrating inferior LRF and survival in HPV+ patients with EGFR overexpression [176,177].

It is likely that data from a much larger patient population would reveal a statistically significant relationship between EGFR overexpression and outcomes in HPV+OPC patients, as a small non-significant trend was observed in some studies reporting lack of statistically significant relationships [82,83]. However, even if found to be statistically significant in a larger cohort, EGFR overexpression is unlikely to be a clinically useful biomarker for risk-stratification in good-prognosis HPV+OPC.

While EGFR mutations may carry a similar adverse prognosis in HNC as overexpression of the gene, they are very rare in HNC [178] and are not likely to affect HPV+OPC management.

#### 3.5.4. HPV Integration into the Host Genome

The HPV genome can exist in an episomal, non-integrated state, or an integrated state, where the virus incorporates all or part of its genome into the host’s genome. Integration often disrupts the E2 gene, which is responsible for regulating E6 and E7, resulting in increase in their expression and leading to greater oncogenic activity and genetic instability [88]. Genomic integration of high-risk HPV has been shown to alter gene expression in oropharyngeal squamous cell carcinoma [89]. HPV integration status has been directly used in stratifying HPV+OPC: Integration-negative patients demonstrated statistically significant improvement in OS and were characterized by heightened signatures for immune cells compared to integration-positive tumors [88,90]. Other studies did not report a statistically significant association of integration status with clinical outcomes [179,180]. Yarbrough et al. commented that all studies using transcriptional (RNAseq) methods to verify integration found it to be associated with poor prognosis [181]. HPV integration sites can be silent, or they can be productive, actively transcribed and leading to the production of viral-host fusion transcripts [182]. Tumors with productive HPV integration are associated with higher E6/E7 proteins and enhanced tumor aggressiveness compared with silent ones. Studies determining the outcomes of productive vs. silent integration need to be made to assess their role in HPV+OPC.

#### 3.5.5. HPV Copy Numbers/Viral Load

The prognostic impact of intratumoral viral copy numbers (“viral load”, VL) has been examined by several groups. Related publications define low, medium, or high VL thresholds based on various statistical analyses. Most reported statistically significant trends for better outcomes related to high VL [91,92,93,94,109], including a large study demonstrating no correlation between tumor stage or smoking status and the VL, suggesting that it may be useful for stratifying good prognostic HPV+OPC [94]. However, while all series demonstrated that low intratumor VL is a detrimental prognostic factor, the thresholds differ markedly among the series, likely depending on the specific method assessing VL, and are therefore not yet ready for clinical decision making.

#### 3.5.6. DNA Methylation

Wild type p53 negatively regulates DNA methyltransferase 1 (DNMT1) expression, and in HPV+OPC the degradation of p53 caused by E6 oncoprotein consequently upregulates DNMT1, causing hypermethylation. HPV+OPC tends therefore to harbor a higher amount of aberrantly methylated DNA than HPV-negative OPC [95]. Aberrant DNA hypermethylation of promoter regions is a major mechanism for silencing tumor suppressor genes affecting cell-cycle regulation, apoptosis, cellular adhesion, cellular migration, and differentiation [95].

Nakagawa et al. demonstrated in a large cohort of OPC patients three distinct methylation groups: high, medium, and low methylation genotypes [96]. All the patients in the high group consisted of HPV+OPC and the highest methylation levels in promoter regions showed the most favorable outcome. A correlation between high methylation state and episomal HPV DNA has been reported, and it is possible that episomal DNA might be involved in DNA methylation induction, associated with favorable prognosis [96].

The Ten Eleven Translocation (TET) enzymes oxidize 5-methylcytosines and promote locus-specific reversal of DNA methylation. The activity of TET enzymes, which are involved in removing epigenetic marks and function as DNA demethylases, is an important tumor suppressor mechanism [183], and promoter methylation of some of the TET genes was associated with worse prognosis in OPC [184]. Thus, the role of methylation in the prognosis of HPV+OPC may depend on the specific genes methylated, whether tumor promoters or suppressors. Current related literature is scant, and the data is not yet sufficient for decision making based on the methylation status of any specific gene.

#### 3.5.7. Estrogen Receptors

Estrogen receptors (ERs) exist in two isoforms, ERα and Erβ. While the role of ERβ in cancer biology is controversial, ERα is as a factor involved in tumorigenesis and cancer progression [71]. Tumors are considered ERα-positive if more than 1% cancer cells show nuclear staining in IHC and the few studies that assessed the prognostic value of Erα in HPV+OPC reported that 40–55% of patients had tumors staining positive, compared with a very low rate of positive staining in HPV-negative OPC [71,72,73]. All these studies reported significant DFS and OS benefit in HPV+OPC patients with Erα positive staining compared with those with negative.

The exact mechanisms by which HPV and estrogen receptors interact are incompletely understood. Studies demonstrated a synergistic effect, with HPV activating ERα response elements, and ERα inducing transcription of the HPV genome [185].

In summary, the relatively few studies assessing the role of Erα in the prognosis of HPV+OPC suggest a potentially substantial survival and DFS benefit for those with positively stained tumors, constituting approximately half of the HPV+OPC patient population. Adding ERα staining to ongoing trials of HPV+OPC may shed light on its potential role in the selection of patients for de-escalated therapy.

#### 3.5.8. NF-Kappa B

Nuclear factor-kappaB (NF-kB), a transcription factor that modulates expression of factors involved in inflammation, immunity, proliferation, and apoptosis, is constitutively activated and plays a role in pathogenesis and therapeutic resistance in HNC. Several studies reported that NF-kB overexpression in OPC is associated with resistance to chemo-radiotherapy and worse prognosis [97]. These earlier reports did not specify HPV status and their patient population likely included mostly smoking and alcohol-related OPC. In contrast to these reports, Schrank et al. reported that patients with HPV+OPC whose tumors harbored constitutively active NF-κB had improved survival in three independent cohorts [98]. NF-κB was the only gene set among many genes analyzed that successfully segregated HPV+OPC patients into prognostic groups associated with differences in DFS, leading to the conclusion that most of the survival benefit of HPV+OPC is attributable to the subtype with highly active NF-κB. The findings that the activity of NF-kB in HPV+OPC is associated with outcomes that are opposite to those reported in older, likely non-HPV HNC series, are intriguing and require extensive validation.

#### 3.5.9. Cyclin D1

Cyclin D1 is a family of proteins regulating the progression of the cell cycle at the G1-S checkpoint. As a result of either gene amplification or rearrangement, Overexpression of cyclin D1 due to gene amplification can contribute significantly to tumor growth. Several studies reported an adverse effect of Cyclin D overexpression on outcomes in HPV+OPC [99,100]. The Washington University group analyzed cyclin D1 immunostaining on tissue microarrays of OPC patients and reported that the intensity of cyclin D1 expression significantly stratified the HPV+OPC patients into prognostic subgroups independent of other variables [100]. 14.4% of HPV+OPC tumors had cyclin D1 expression > 10% and 2.7% had expression > 30%. As the staining intensity of cyclin D1 may stratify HPV+OPC patients into clinically different groups, it complicates its potential application in routine clinical practice. Also, significant differences were found in average cyclin D1 positivity among multi-site blocks within the same tumor [186], increasing the uncertainties in the potential use of Cyclin D1 expression in decision-making.

#### 3.5.10. Ki67

Ki67 antigen is expressed during the active phases of the cell cycle, a marker of proliferative capacity and a poor prognostic factor in several cancers, including head and neck squamous cell carcinoma. Very few studies assessed its prognostic effect in HPV+OPC, reporting better survival in patients with low expression of Ki67 [102,103,104]. However, the small patient numbers and the various Ki67 expression level thresholds in these studies preclude clinical utilization of these data.

#### 3.5.11. Tumor Genomic Heterogeneity

Intratumoral genomic heterogeneity and copy number variations (CNV), common mutations where a chromosome region is either duplicated or deleted, have predicted poor outcomes in many cancers. Recent advances in HPV whole genome sequencing (WGS) technology allow the HPV16 genome to be interrogated at a higher level of detail than previous methods. The Pittsburgh group [58] conducted a large WGS study to characterize the genetic variation in the HPV genome. A total of 284 HPV16 single-nucleotide polymorphisms (SNPs), a variation in a single DNA base pair where one nucleotide is substituted for another, were identified. Eight of these SNPs were found to be significantly associated with worse survival, with a combined prevalence of 15%. Median OS for patients with at least one high-risk SNP was only 3.96 years, compared with 18.67 years for patients without a high-risk HPV16 SNP (*p* < 0.001). As WGS is not routinely used in the clinic, future adoption of WGS in the clinic will verify whether genomic heterogeneity can be used to select poor prognosis HPV+OPC patients.

#### 3.5.12. PIK3CA Mutations

PIK3CA (phosphatidylinositol-4,5-bisphosphate 3-kinase catalytic subunit alpha) gene-coded PI3-K/AKT is a major downstream target of the HER2 tyrosine-kinase receptor family. This pathway is frequently overactivated triggering cell growth and survival and promoting tumor progression. PIK3CA is the most mutated gene in HPV+OPC, carried by 20–30% of patients. In a prospective study of de-intensified chemo-RT in HPV+OPC, the UNC group reported that patients with wild-type-PIK3CA had statistically significantly higher 3-year DFS than PIK3CA-mutant patients [74]. On MVA, PIK3CA mutation was the only variable statistically significantly associated with disease recurrence. This group suggested excluding these patients from future de-escalating trials. In contrast, several groups reported either no difference in outcome of HPV+OPC with mutated or wild-type PIK3CA [75] or even better outcomes in patients with aberrations in the PI3k pathway or PIK3CA mutations [76,77,78]. Notwithstanding the fact that the study reporting the adverse effect of mutations was the largest and the only prospective one [75], the contradicting results reported by others regarding the direction of the prognostic effects of PIK3CA mutations preclude their current clinical use.

#### 3.5.13. Chromosome 3p Deletion

Chromosome 3p is a specific region on chromosome 3 containing tumor suppressor genes. The loss of the chromosome 3p arm is frequently associated with TP53 mutations, leading to poor prognosis [101]. As HPV+OPC rarely contains TP53 mutations, the prognostic effect of an isolated 3p deletion has been questioned. In an analysis of 59 HPV+OPC tumors, the 25 tumors with 3p deletion had significantly worse prognosis than 34 without the 3p event [187]. Similarly, the MSKCC group reported a significant enrichment for 3p deletion (9.1-fold; *p* = 0.002) in recurrent and metastatic HPV+OPC compared with primary HPV+OPC tumors [187]. The MSKCC group later performed a validation study and included an independent cohort reporting no survival differences between 3p arm loss and intact tumor cohorts [188]. Based on the results of the external validation study, the authors concluded that 3p arm status may not be a useful biomarker of survival in HPV+OPC and noted the differences between the studies regarding how HPV+ and 3p arm loss were determined. This negative study emphasizes the need for external validation which is missing in most related studies.

#### 3.5.14. Micro RNAs

MicroRNAs (miRNAs) are small noncoding RNAs involved in the regulation of most protein-coding genes, are frequently deregulated, and can potentially be used as prognostic biomarkers. Very few studies have assessed their prognostic value in HPV+OPC. The Washington University group sequenced and identified 620 known miRNAs from two OPC cohorts [85]. A 26-miRNA signature was identified and was prognostic when applied to HPV+ patients, maintaining its independent prognostic value on MVA, However, the authors noted that many of the selected miRNAs had no known biological function, limiting their potential clinical application. A smaller study of 56 HPV+OPC patients found 224 miRNAs expressed in most samples, with 5 of these significantly associated with better outcomes [86]. Only a part of these miRNAs overlapped with the 26 miRNAs found to be prognostic in the Washington University study.

The uncertainties related to the prognostic utility of miRNA in HPV+OPC exist in other tumor types. For example, two meta-analyses of the effects of miR-200 on the prognosis of various tumors have reached opposite conclusions [87,189].

### 3.6. Metabolomics and Proteomics

The hypothesis that metabolism-related gene expression differences unique to HPV+OPC may influence patient survival was tested by the University of Western Ontario group [190]. They analyzed 229 metabolic genes and identified numerous differentially expressed genes between HPV+OPC and HPV-negative OPC patients. Reduced expression of seven metabolism-related genes was correlated with improved survival in the HPV+ group, suggesting that transcriptional alterations in metabolic genes may serve as predictive biomarkers of outcome. This study used multiple comparisons, defining statistically significant expressions at *p* ≤ 0.05 without correction.

Proteomic signatures that may stratify treatment response were studied [105]. Forty HPV+OPC tumor samples (20 disease-free and 20 recurrent) were surveyed. Of 1414 quantified proteins, 77 demonstrated significant differential expression between recurrent and non-recurrent tumors. Top enriched functional pathways in the recurrent cohort included pathways involved in programmed cell death and apoptosis, each consisting of 73 proteins, while similar numbers of other pathways were significantly downregulated in the recurrent cohort. The authors discussed several issues that restricted the generalizability of their findings, especially the small cohort size that precluded their ability to validate the chosen pathways and limited their ability to identify other proteomic targets associated with recurrent tumors.

## 4. Discussion

Based on the results of randomized studies of treatment de-escalation of clinically good prognosis HPV+OPC patients (having an early-stage disease and no or minimal smoking history), approximately 10–15% of these patients have worse outcomes and should be excluded from this treatment strategy. An effective way to identify such patients before or early during therapy is needed. Our review assessed two major potential selection methods: Based on established poor-prognosis biomarkers of HPV+OPC, and an empiric method relying on tumor response to induction chemotherapy or the response of its hypoxic, metabolic, and genomic parameters during the initial phase of the definitive therapy.

While the need to verify HPV positivity by DNA or RNA PCR in addition to p16 immunohistochemistry alone, which has up to 15% false positivity [27] is obvious, a selection based on known, previously published poor-prognosis biomarkers is attractive and has a strong rationale. Many such biomarkers were found in the literature and discussed in this paper. However, besides factors like iENE which is consistently associated with poor prognosis in HPV+OPC, we have found many factors hindering this approach. One factor is the relatively high prevalence, up to 50% of the patient cohorts, of some of the adverse-prognosis biomarkers which preclude each from being a sole selecting factor. Examples include tumor stem cell markers [66], estrogen receptor-negative tumors [71], and others, suggesting that their use would require one or more additional selection methods. On the other hand, some adverse prognostic markers are very rare in HPV+OPC, making their use obsolete, such as disruptive TP53 mutations [80] and EGFR mutations or overexpression [83]. Most studies were small and lacked internal or external validation. In many studies there were relatively small numbers of patients compared with the number of variables tested. All retrospective studies and most prospective non-randomized studies had fair or poor NOS scores, mostly due to lack of sufficient f/u and lack of information about loss to follow-up. Consequently, we have found conflicting and contradicting reports of the effects of many of the potential prognostic markers on the outcomes of HPV+OPC, hampering the utility of these markers in decision-making. These limitations are widespread: studies with methodological flaws and small studies may overestimate treatment effects and contribute to statistical heterogeneity [106]. For example, Dhawan et al. assessed clinico-pathological gene signature predictors in published series of OPC [53]. Gene sets often could be identified with strong correlation to phenotypes using single datasets, but many of these signatures became less cohesive and lost the power to predict phenotypes when applied to external datasets.

Efforts to implement predictive biomarkers are hampered also by intratumor heterogeneity that is not represented by a single tumor biopsy or FNA [191]. While “liquid biopsy” may reveal genetic and epigenetic variabilities through plasma ctDNA, TCs, or EVs, our review found that this strategy using clinically available tools like PCR may not be sensitive enough in HPV+OPC due to the low likelihood of the detection of mutational variants [67,68]. However, emerging results of newer technology like NGS are encouraging [69].

A specific limitation of assessing adverse prognostic biomarkers in good-prognosis HPV+OPC is small number of failures in these patients, requiring large multicenter trials. Apart from conducting large enough prospective studies with internal and external validation cohorts, models need to consider multiple biological prognostic candidates in addition to the clinical variables, as a single prognostic biomarker is unlikely to predict most of the patients at risk [192].

The alternative selection methods are empiric, entailing selection of patients whose tumors respond well early in therapy. This includes observing the response to induction chemotherapy and proceeding with de-escalated definitive treatment only in patients with a pre-defined substantial response. This approach has been successful in several phase II studies but must take into consideration the added acute toxicity associated with induction, which competes with the potential benefits of the de-escalation of the definitive therapy, and the lack of survival benefit of induction chemotherapy in OPC [31].

Compelling approaches have been using the kinetics of pre-treatment tumor hypoxia (MSKCC group), metabolic activity (Michigan group), or ctDNA counts (UNC group and others) during the early phase of the definitive therapy to select patients with responding tumors for de-escalation. Multi-institutional randomized studies comparing response-based de-escalation strategies to SOC are required to assess whether these strategies are non-inferior to SOC.

In recent years, machine learning (ML) and deep learning (DL) techniques, subfields of Artificial Intelligence (AI), have shown promising results in prognostication efforts in HNC due to their ability to establish complex relationships between datasets and to classify different patterns to predict outcomes [193]. AI has enhanced the analysis of clinical [141], molecular [194], radiological [195], and histological [196] features in predictive models in OPC. A potential important benefit of AI is its ability to integrate information using different prognostic markers in predictive models [52,195].

The challenges and limitations in implementing AI models include a lack of standardized data collection, variations in AI model development and generation, low-quality evidence on model performance, and a lack of internal and external validation [193,197]. While the initial reports suggest a substantial benefit from the opportunities offered by AI, internal and external validation of AI-derived results in large prospective studies are still required before their clinical adoption.

Due to multiple shortcomings of the studies of prognostic biomarkers of HPV+OPC reviewed in this paper, using them for the selection of favorable-prognosis HPV+OPC patients who may not benefit from treatment de-escalation is not feasible at this time. The data presented in this study, as well as the results of the randomized studies which demonstrated a lack of non-inferiority of de-escalation to SOC, emphasize the consensus that de-escalation of therapy for good-prognosis HPV+OPC patients should not be done in the clinic outside of a careful clinical study [198]. Most compelling currently are developing and validating response-adapted strategies. Immediate promise lies in leveraging widely available tools like FDG-PET and ctDNA to guide adaptive therapy within randomized trials, which should be large enough to ensure the power required to validate their outcomes. Simultaneously, concerted efforts to standardize and validate multi-modal AI models are essential for the next generation of precision de-escalation.

Whether treated with de-escalated or standard therapy, RT planning aiming to minimize long-term late toxicity, notably xerostomia and dysphagia, must be implemented, as prior studies have shown that long-term quality of life (QOL) can be preserved even following a full chemo-RT course [199]. These standards will hopefully be eclipsed by successful selections of patients for treatment de-escalation.

In conclusion, our results support the consensus that de-escalation of the therapy of good-prognosis HPV+OPC should not be done outside of clinical studies, and that large multi-institutional studies are required to assess the utility of any biomarker or strategy aiming for patient selection of de-escalated therapy.

## Figures and Tables

**Table 1:** Empirical approaches and prognostic biomarkers in HPV-positive oropharyngeal cancer.

Biomarker Category	Biomarker	Specific Biomarker(s)	Prognostic Significance	Key Limitations/Notes	Key References
**HPV verification**	HPV verification in addition to p16	p16 IHC + HPV DNA/RNA PCR	Improves risk stratification; discordant p16+/HPV− worse	p16 alone: up to ~15% false positives	[27]
**Empirical: tumor response-based** **decisions**	Response to induction chemotherapy	PR or CR following induction	Consistent favorable outcomes of cancers responding to induction vs. non-responders	Toxicity of induction; No evidence of improved OS in OPC	[4,28,29,30,31]
	Hypoxia PET imaging pre and during therapy	FMISO-PET, FDG PET, FET NIM PET, FAZA PET pre and during therapy	Absence/ clearance of hypoxia → excellent outcomes	Limited clinical availability of hypoxia imaging	[32]
	FDG-PET imaging pre and during therapy	Baseline MTV and MTV response at 2 weeks during chemo-RT	Limited baseline MTV, and MTV reduction by >50% from baseline → improved outcomes	FDG-PET widely clinically available	[33,34]
	Ct HPVDNA pre and during therapy	Ct HPV DNA pre therapy and at 4 weeks of therapy	Low ctDNA favorable; Clearance at week 4: very favorable	Different methods detecting ctDNA and different thresholds in studies	[35,36,37]
**Clinical, radiological, and histological biomarkers**	Radiomics	CT/MRI/PET features	Variable prognostic value	Conflicting studies, various modeling and methodologies	[38,39]
	Histopathology morphology	Anaplasia, multinucleation	Higher—worse prognosis	Conflicting reports	[40,41]
	Histopathology image analysis	Spatial histological features	Homogeneity—favorable	Conflicting reports, lack of standard algorithms	[41,42]
	Imaging-based extracapsular extension	MRI and CT based, in the absence of matted nodes	Worse prognosis	Requires MRI, CT, and neuroradiologist for best detection	[43,44,45,46,47]
**Immune-related** **biomarkers**	Neutrophil/lymphocyte ratio	Circulating lymphocyte/neutrophil ratio	Higher ratio—favorable	Large interindividual variations, no clear threshold	[48,49,50]
	Tumor infiltrating lymphocytes (TILs)	TILs in tumor and stroma	High TILs in tumor and stroma better	Different scoring methods and results in the literature	[51,52]
	Immune vs. keratinized TME; tumor to stroma ratio	Immune-rich vs. keratinized TME characteristics	Immune—better	Keratinized rare in HPV+OPC, associated with smoking	[53,54]
	HPV-related proteins and their antibodies	Primarily E6, E7	Conflicting reports	The role of E6 isoform needs evaluation	[55,56,57,58,59,60,61,62]
	PD-L1 levels	Tumor score or combined tumor and lymphocyte score (%)	Prognostic in patients receiving immunotherapy, less clear in chemo-RT	Requires more data, especially for combined score ≤ 1%	[63,64,65]
**Molecular biomarkers**	Cancer stem cell markers	CD44, MET, ALDH1A1, BMI1	High expression = adverse outcomes	High prevalence in HPV+OPC; thresholds and prognostic variabilities	[65,66]
	“liquid biopsy”	Mutational and epigenetic variants in ctHPV DNA, CTCs, exosomes	Currently no data	Needs whole-genome next-generation sequencing to detect, low abundance of CTCs, no validated bioactive exosomes	[67,68,69,70]
	Estrogen receptors	ERα	ERα+ → better DFS/OS	Very few studies	[71,72,73]
	PIK3CA mutations	PIK3CA hotspot mutations	Conflicting prognostic data	Requires validation	[74,75,76,77,78]
	TP53 disruptive mutations	TP53	Poor prognosis in HPV-unrelated OPC	Very rare in HPV+OPC	[79,80]
	EGFR overexpression or mutations	EGFR	Worse prognosis in HPV-unrelated OPC	Very rare in HPV+OPC	[18,81,82,83,84]
	Chromosomal alterations	Chromosome 3p arm deletion	Early reports worse; newer data no effect	Conflicting evidence	[85]
	MicroRNA signatures	miRNA signature	Better or worse prognostic depending on individual miRNAs	Few studies; no consistency in the specific prognostic miRNAs	[86,87]
	HPV episomal vs. integrated genome	Genomic integration of HPV genome	Conflicting reports	Need to assess productive vs. silent integration	[88,89,90]
	HPV copy number/viral load	Intratumoral HPV genome copy numbers ratio to a diploid control gene	Low load-worse prognosis	Thresholds depend on the specific assessment method	[91,92,93,94]
	DNA methylation	Aberrantly epigenetic methylated DNA	Depends on which gene is methylated (better prognosis if methylation of promoter rather than suppressor gene)	Scant data in the literature	[95,96]
	NF-kappa B transcription factor	Increased transcriptional activity	Usually poor prognostic in HNC (HPV−), but reported good prognosis in one HPV+OPC study	Requires validation in additional HPV+OPC studies	[97,98]
	Cyclin D1	Overexpression	Poor prognosis	Outcomes depend on staining intensity of cyclin D; no clear threshold	[99,100]
	HPV tumor genomic heterogeneity	Copy number variations (CNVs), single-nucleotide poly morphism (SNP)	High risk SNP: worse outcomes	Requires whole genome sequencing; not routinely used in the clinic	[74]
	PICK3A	Mutation	Conflicting data regarding prognosis	Conflicting data precludes current clinical use	[77,78,101]
	Ki 67	Antigen expressed during cell proliferation	Higher expression—poorer outcomes	Few studies on HPV+OPC, Small patient numbers and various expression level thresholds	[102,103,104]
**Metabolic and proteomic biomarkers**	Metabolic gene expression	7 specific metabolic downregulated genes	Low expression—improved survival	Exploratory study	[105]
	Proteomic panels	77 specific proteins	Correlate with recurrence	Small sample; no validation	[106]

## Data Availability

No new data were created or analyzed in this study. Data sharing is not applicable to this article.

## References

[1] Gillison M.L., Chaturvedi A.K., Anderson W.F., Fakhry C. (2015). Epidemiology of Human Papillomavirus-Positive Head and Neck Squamous Cell Carcinoma. J. Clin. Oncol..

[2] Ang K.K., Harris J., Wheeler R., Weber R., Rosenthal D.I., Nguyen-Tân P.F., Westra W.H., Chung C.H., Jordan R.C., Lu C. (2010). Human papillomavirus and survival of patients with oropharyngeal cancer. N. Engl. J. Med..

[3] Gillison M.L., D’Souza G., Westra W., Sugar E., Xiao W., Begum S., Viscidi R. (2008). Distinct risk factor profiles for human papillomavirus type 16-positive and human papillomavirus type 16-negative head and neck cancers. J. Natl. Cancer Inst..

[4] Chen A.M., Felix C., Wang P.-C., Hsu S., Basehart V., Garst J., Beron P., Wong D., Rosove M.H., Rao S. (2017). Reduced-dose radiotherapy for human papillomavirus-associated squamous-cell carcinoma of the oropharynx: A single-arm, phase 2 study. Lancet Oncol..

[5] Misiukiewicz K., Gupta V., Miles B.A., Bakst R., Genden E., Selkridge I., Surgeon J.T., Rainey H., Camille N., Roy E. (2019). Standard of care vsreduced-dose chemoradiation after induction chemotherapy inHPV+ oropharyngeal carcinoma patients: The quarterback trial. Oral Oncol..

[6] Chera B.S., Amdur R.J., Tepper J.E., Tan X., Weiss J., Grilley-Olson J.E., Hayes D.N., Zanation A., Hackman T.G., Patel S. (2018). Mature results of a prospective study of deintensified chemoradiotherapy for low-risk human papillomavirus-associated oropharyngeal squamous cell carcinoma. Cancer.

[7] Yom S.S., Torres-Saavedra P., Caudell J.J., Waldron J.N., Gillison M., Xia P., Truong M.T., Kong C., Jordan R., Subramaniam R.M. (2021). Reduced-dose radiation therapy for HPV-associated oropharyngeal carcinoma (NRG Oncology HN002). J. Clin. Oncol..

[8] Takemoto N., Seo Y., Nakahara S., Yoshyoka Y., Teshima T., Fujii T., Isohashi F., Otani K., Takenaka Y., Suzuki M. (2021). Radiation therapy alone for human papillomavirus-related squamous cell carcinoma of the oropharynx: A single-arm, phase 2 study. Int. J. Radiat. Oncol. Biol. Phys..

[9] Rosenthal D., Harari P.M., Giralt J., Bell D., Raben D., Liu J., Schulten J., Ang K.K., Bonner J.A. (2016). Association of Human Papillomavirus and p16 Status with Outcomes in the IMCL-9815 Phase III Registration Trial for Patients with Locoregionally Advanced Oropharyngeal Squamous Cell Carcinoma of the Head and Neck Treated with Radiotherapy with or Without Cetuximab. J. Clin. Oncol..

[10] Tam M., Riaz N., Kannarunimit D., Peña A.P., Schupak K.D., Gelblum D.Y., Wolden S.L., Rao S., Lee N.Y. (2015). Sparing bilateral neck level IB in oropharyngeal carcinoma and xerostomia outcomes. Am. J. Clin. Oncol..

[11] Gutiontov S., Leeman J., Lok B., Romesser P., Riaz N., Tsai C.J., Lee N., McBride S. (2016). Cervical nodal level V can safely be omitted in the treatment of locally advanced oropharyngeal squamous cell carcinoma with definitive IMRT. Oral Oncol..

[12] Leeman J.E., Gutiontov S., Romesser P., McBride S., Riaz N., Lee N., Tsai C.J. (2017). Sparing of high retropharyngeal nodal basins in patients with unilateral oropharyngeal carcinoma treated with intensity modulated radiation therapy. Pract. Radiat. Oncol..

[13] Deschuymer S., Nevens D., Duprez F., Daisne J.F., Dok R., Laenen A., Voordeckers M., De Neve W., Nuyts S. (2020). Randomized clinical trial on reduction of radiotherapy dose to the elective neck in head and neck squamous cell carcinoma; update of the long-term tumor outcome. Radiother. Oncol..

[14] Sher D.J., Pham N.L., Shah J.L., Sen N., Williams K.A., Subramaniam R.M., Moore W., Chorley R., Ahn C., Khan S.M. (2021). Prospective phase 2 study of radiation therapy dose and volume de-escalation for elective neck treatment of oropharyngeal and laryngeal cancer. Int. J. Radiat. Oncol. Biol. Phys..

[15] Tsai C.J., McBride S.M., Riaz N., Kang J.J., Spielsinger D.J., Waldenberg T., Gelblum D., Yu Y., Chen L.C., Zakeri K. (2022). Evaluation of substantial reduction in elective radiotherapy dose and field in patients with Human papillomavirus-associated oropharyngeal carcinoma treated with definitive chemoradiotherapy. JAMA Oncol..

[16] Gillison M.L., Trotti A.M., Harris J., Eisbruch A., Harari P.M., Adelstein D.J., Jordan R.C.K., Zhao W., Sturgis E.M., Burtness B. (2018). Radiotherapy plus cetuximab or cisplatin in human papillomavirus-positive oropharyngeal cancer (NRG Oncology RTOG 1016): A randomised, multicentre, non-inferiority trial. Lancet.

[17] Mehanna H., Robinson M., Hartley A., Kong A., Foran B., Fulton-Lieuw T., Dalby M., Mistry P., Sen M., O’Toole L. (2019). Radiotherapy plus cisplatin or cetuximab in low-risk human papillomavirus-positive oropharyngeal cancer (De-ESCALaTE HPV): An open-label randomised controlled phase 3 trial. Lancet.

[18] Rischin D., King M., Kenny L., Porceddu S., Wratten C., Macann A., Jackson J.E., Bressel M., Herschtal A., Fisher R. (2021). Randomized trial of radiation therapy with weekly cisplatin or cetuximab in low-risk HPV-associated oropharyngeal cancer (TROG12.01). Int. J. Radiat. Oncol. Biol. Phys..

[19] Gebre-Medhin M., Brun E., Engström P., Haugen Cange H., Hammarstedt-Nordenvall L., Reizenstein J., Nyman J., Abel E., Friesland S., Sjödin H. (2021). ARTSCAN III: A randomized phase III study comparing chemoradiotherapy with Cisplatin versus Cetuximab in patients with locoregionally advanced head and neck squamous cell cancer. J. Clin. Oncol..

[20] Yom S.S., Harris J., Caudell J.J., Geiger J.L., Waldron J., Gillison M., Subramanian R.M., Yao M., Xiao C., Kovalchuk N. (2024). Interim Futility Results of NRG-HN005, A Randomized, Phase II/III Non-Inferiority Trial for Non-Smoking p16+ Oropharyngeal Cancer Patients. Int. J. Radiat. Oncol. Biol. Phys..

[21] Petrelli F., Luciani A., Ghidini A., Cherri S., Gamba P., Maddalo M., Bossi P., Zaniboni A. (2022). Treatment de-escalation for HPV+ oropharyngeal cancer: A systematic review and meta-analysis. Head Neck.

[22] Price K.A.R., Nichols A.C., Shen C.J. (2020). Novel Strategies to Effectively De-escalate Curative-Intent Therapy for Patients with HPV-Associated Oropharyngeal Cancer: Current and Future Directions. Am. Soc. Clin. Oncol. Educ. Book.

[23] Chen A.M. (2024). Translational risk-adapted approaches to de-escalated radiation for human papillomavirus-positive oropharyngeal cancer: Past, present, and future. Oral Oncol..

[24] Hopkins D. (2025). The role of biomarkers in the management of HPV-related oropharyngeal cancer. Curr. Opin. Oncol..

[25] Lim Y., Mierzwa M., Sartor M.A. (2023). Clinical, morphologic and molecular heterogeneity of HPV-associated oropharyngeal cancer. Oncogene.

[26] Wells G.A., Shea B., O’Connell D., Peterson J., Welch V., Losos M. The Newcastle-Ottawa Scale (NOS) for Assessing the Quality of Nonrandomised Studies in Meta-Analyses. http://www.ohri.ca/programs/clinical_epidemiology/oxford.asp.

[27] Seiwert T.Y. (2014). Ties that bind: P16 as a prognostic biomarker and the need for high accuracy. J. Clin. Oncol..

[28] Marur S., Li S., Cmelak A.J., Zhao W.J., Ferris R.L., Westra W.H., Gilbert J., Bauman J.E., Wagner L.I., Trevarthen D.R. (2017). E1308: Phase II trial of induction chemotherapy followed by reduced-dose radiation and weekly cetuximab in patients with HPVassociated resectable squamous cell carcinoma of the oropharynx—ECOG-ACRIN Cancer Research Group. J. Clin. Oncol..

[29] Seiwert T.Y., Foster C.C., Blair E.A., Karrison T.G., Agrawal N., Melotek J.M., Portugal L., Brisson R.J., Dekker A., Kochanny S. (2019). OPTIMA: A phase II dose and volume de-escalation trial for human papillomavirus-positive oropharyngeal cancer. Ann. Oncol..

[30] Rosenberg A.J., Agrawal N., Juloori A., Cursio J., Gooi Z., Blair E., Chin J., Ginat D., Pasternak-Wise O., Hasina R. (2024). Neoadjuvant Nivolumab plus chemotherapy followed by response-adaptive therapy for HPV+ oropharyngeal cancer: OPTIMA II phase 2 open-label nonrandomized controlled trial. JAMA Oncol..

[31] Sher D.J., Schwartz D.L., Nedzi L., Khan S., Hughes R., Fidler M.J., Koshy M. (2016). Comparative effectiveness of induction chemotherapy for oropharyngeal squamous cell carcinoma: A population-based analysis. Oral Oncol..

[32] Lee N.Y., Sherman E.J., Sch€oder H., Wray R., Boyle J.O., Singh B., Grkovski M., Paudyal R., Cunningham L., Zhang Z. (2024). Hypoxia-directed treatment of Human papillomavirus-related oropharyngeal carcinoma. J. Clin. Oncol..

[33] Allen S.G., Rosen B.S., Aryal M., Cao Y., Schipper M.J., Wong K.K., Casper K.A., Chinn S.B., Malloy K.M., Prince M.E. (2023). Initial Feasibility and Acute Toxicity Outcomes From a Phase 2 Trial of ^18^F-Fluorodeoxyglucose Positron Emission Tomography Response-Based De-escalated Definitive Chemoradiotherapy for p16+ Oropharynx Cancer: A Planned Interim Analysis. Int. J. Radiat. Oncol. Biol. Phys..

[34] Regan S.N., Cao Y., Allen S.G. (2024). 16-FDG-PET-based selective de-escalation of radiotherapy for HPV-related oropharynx cancer: Results form a phase II trial. 2024 Multidisciplinary Head Current Oncology Reports and Neck Cancers Symposium. Int. J. Radiat. Oncol. Biol. Phys..

[35] Chera B.S., Kumar S., Beaty B.T., Marron D., Jefferys S., Green R., Goldman E.C., Amdur R., Sheets N., Dagan R. (2019). Rapid clearance profile of plasma circulating tumor HPV type 16 DNA during chemoradiotherapy correlates with disease control in HPV-associated oropharyngeal cancer. Clin. Cancer Res..

[36] Haring C.T., Dermody S.M., Yalamanchi P., Kang S.Y., Old M.O., Chad Brenner J., Spector M.E., Rocco J.W. (2022). The future of circulating tumor DNA as a biomarker in HPV related oropharyngeal squamous cell carcinoma. Oral Oncol..

[37] Adrian G., Forslund O., Pedersen L., Sjövall J., Gebre-MedhinM (2023). Circulating tumour HPV16 DNA quantification—A prognostic tool for progression-free survival in patients with HPV-related oropharyngeal carcinoma receiving curative chemoradiotherapy. Radiother. Oncol..

[38] Araújo A.L.D., Kowalski L.P., Santos-Silva A.R., Louredo B.V.R., Saldivia-Siracusa C., de Melo O.A.A.M., Cabral D., Coca-Pelaz A., Guntinas-Lichius O., de Bree R. (2026). Radiomic-Based Machine Learning Classifiers for HPV Status Prediction in Oropharyngeal Cancer: A Systematic Review and Meta-Analysis. Diagnostics.

[39] Spadarella G., Ugga L., Calareso C., Villa R., D’Aniello S., Cuocolo R. (2022). The impact of radiomics for human papillomavirus status prediction in oropharyngeal cancer: Systematic review and radiomics quality score assessment. Neuroradiology.

[40] Lewis J.S., Ali S., Luo J., Thorstad W.L., Madabhushi A. (2014). A quantitative histomorphometric classifier (QuHbIC) identifies aggressive versus indolent p16-positive oropharyngeal squamous cell carcinoma. Am. J. Surg. Pathol..

[41] Klein S., Quaas A., Quantius J., Löser H., Meinel J., Peifer M., Wagner S., Gattenlöhner S., Wittekindt C., von Knebel Doeberitz M. (2021). Deep learning predicts HPV association in oropharyngeal squamous cell carcinomas and identifies patients with a favorable prognosis using regular H&E stains. Clin. Cancer Res..

[42] Thompson L.D.R., Burchette R., Iganej S., Bhattasali O. (2020). Oropharyngeal squamous cell carcinoma in 390 patients: Analysis of clinical and histological criteria which significantly impact outcome. Head Neck Pathol..

[43] Vainshtein J.M., Spector M.E., Ibrahim M., Bradford C.R., Wolf G.T., Stenmark M.H., Worden F.P., McHugh J.B., Prince M.E., Carey T. (2016). Matted nodes: High distant-metastasis risk and a potential indication for intensification of systemic therapy in human papillomavirus-related oropharyngeal cancer. Head Neck.

[44] Benchetrit L., Torabi S.J., Givi B., Haughey B., Judson B.L. (2021). Prognostic Significance of Extranodal Extension in HPV-Mediated Oropharyngeal Carcinoma: A Systematic Review and Meta-analysis. Otolaryngol. Head Neck Surg..

[45] Huang S.H., O’Sullivan B., Su J., Bartlett E., Kim J., Waldron J.N., Ringash J., de Almeida J.R., Bratman S., Hansen A. (2020). Prognostic importance of radiologic extranodal extension in HPV-positive oropharyngeal carcinoma and its potential role in refining TNM-8 cN-classification. Radiother. Oncol..

[46] Huang S.H., Su J., Koyfman S.A., Hoebers F., Bahig H., Yu E., Bartlett E., Spreafico A., Lee J., Stock S. (2025). A Proposal for HPV-Associated Oropharyngeal Carcinoma in the Ninth Edition Clinical TNM Classification. JAMA Otolaryngol. Head Neck Surg..

[47] Mehanna H., Abou-Foul A.K., Henson C., Kristunas C., Nankivell P.C., McDowell L., Leemans C.R., van den Brekel M.W.M., von Buchwald C., Jakobsen K.K. (2025). Accuracy and Prognosis of Extranodal Extension on Radiologic Imaging in Human Papillomavirus-Mediated Oropharyngeal Cancer: A Head and Neck Cancer International Group (HNCIG) Real-world Study. Int. J. Radiat. Oncol. Biol. Phys..

[48] Hughes A., Johnny C.A., Huang S.H., Su J., Bratman S., Cho J., Hahn E., Hosni A., Hope A., Kim J. (2025). The prognostic and predictive value of pre-treatment hematologic markers for oropharyngeal carcinoma stratified by HPV status and treated with definitive (chemo) radiation. Radiother. Oncol..

[49] Price J.M., Mistry H.B., Betts G., Cheadle E.J., Dixon L., Garcez K., Illidge T., Iyizoba-Ebozue Z., Lee L.W., McPartlin A. (2022). Pretreatment Lymphocyte Count Predicts Benefit From Concurrent Chemotherapy with Radiotherapy in Oropharyngeal Cancer. J. Clin. Oncol..

[50] Young C.A., Murray L.J., Karakaya E., Thygesen H.H., Sen M., Prestwich R. (2014). The Prognostic Role of the Neutrophil-to-Lymphocyte Ratio in Oropharyngeal Carcinoma Treated with Chemoradiotherapy. Clin. Med. Insights Oncol..

[51] Succaria F., Kvistborg P., Stein J.E., Engle E.L., McMiller T.L., Rooper L.M., Thompson E., Berger A.E., van den Brekel M., Zuur C.L. (2021). Characterization of the tumor immune microenvironment in human papillomavirus-positive and -negative head and neck squamous cell carcinomas. Cancer Immunol. Immunother..

[52] Nakkireddy S.R., Jang I., Kim M., Yin L.X., Rivera M., Garcia J.J., Bartemes K.R., Routman D.M., Moore E.J., Abdel-Halim C.N. (2024). Integrative analysis of H&E and IHC identifies prognostic immune subtypes in HPV related oropharyngeal cancer. Commun. Med..

[53] Dhawan A., Scott J., Sundaresan P., Veness M., Porceddu S., Hau E., Harris A.L., Buffa F.M., Gee H.E. (2020). Role of gene signatures combined with pathology in classification of oropharynx head and neck cancer. Sci. Rep..

[54] Cavalieri S., Serafini M.S., Carenzo A., Canevari S., Brakenhoff R.H., Leemans C.R., Nauta I.H., Hoebers F., van den Hout M.F.C.M., Scheckenbach K. (2021). Clinical Validity of a Prognostic Gene Expression Cluster-Based Model in Human Papillomavirus–Positive Oropharyngeal Carcinoma. JCO Precis. Oncol..

[55] Della Fera A.N., Warburton A., Coursey T.L., Khurana S., McBride A.A. (2021). Persistent human papillomavirus infection. Viruses.

[56] Anderson K.S., Gerber J.E., D’Souza G., Pai S.I., Cheng J.N., Alam R., Kesiraju S., Chowell D., Gross N.D., Haddad R. (2015). Biologic predictors of serologic responses to HPV in oropharyngeal cancer: The HOTSPOT study. Oral Oncol..

[57] Dahlstrom K.R., Anderson K.S., Cheng J.N., Chowell D., Li G., Posner M., Sturgis E.M. (2015). HPV serum antibodies as predictors of survival and disease progression in patients with HPV-positive squamous cell carcinoma of the oropharynx. Clin. Cancer Res..

[58] Lang Kuhs K.A., Faden D.L., Chen L., Smith D.K., Pinheiro M., Wood C.B., Davis S., Yeager M., Boland J.F., Cullen M. (2022). Genetic variation within the human papillomavirus type 16 genome is associated with oropharyngeal cancer prognosis. Ann. Oncol..

[59] Fakhry C., Qualliotine J.R., Zhang Z., Agrawal N., Gaykalova D.A., Bishop J.A., Subramaniam R.M., Koch W.M., Chung C.H., Eisele D.W. (2016). Serum antibodies to HPV16 early proteins warrant investigation as potential biomarkers for risk stratification and recurrence of HPV-associated oropharyngeal cancer. Cancer Prev. Res..

[60] Zhang Y., Waterboer T., Haddad R.I., Miles B.A., Wentz A., Gross N.D., Fakhry C., Quon H., Lorch J.H., Gourin C.G. (2017). Human papillomavirus (HPV) 16 antibodies at diagnosis of HPV-related oropharyngeal cancer and antibody trajectories after treatment. Oral Oncol..

[61] Spector M.E., Sacco A.G., Bellile E., Taylor J.M.G., Jones T., Sun K., Brown W.C., Birkeland A.C., Bradford C.R., Wolf G.T. (2017). E6 and E7 Antibody Levels Are Potential Biomarkers of Recurrence in Patients with Advanced-Stage Human Papillomavirus-Positive Oropharyngeal Squamous Cell Carcinoma. Clin. Cancer Res..

[62] Qin T., Koneva L.A., Liu Y., Zhang Y., Arthur A.E., Zarins K.R., Carey T.E., Chepeha D., Wolf G.T., Rozek L.S. (2020). Significant association between host transcriptome-derived HPV oncogene E6* influence score and carcinogenic pathways, tumor size, and survival in head and neck cancer. Head Neck.

[63] Polesei J., Menegaldo A., Tirelli G., Giacomarra V., Guerrieri R., Baboci L., Casarotto M., Lupato V., Fanetti G., Boscolo-Rizzo P. (2021). Prognostic Significance of PD-L1 Expression In Patients with Primary Oropharyngeal Squamous Cell Carcinoma: A Meta-Analysis. Front. Oncol..

[64] Solomon B., Young R.J., Bressel M., Urban D., Hendry S., Thai A., Angel C., Haddad A., Kowanetz M., Fua T. (2018). Prognostic Significance of PD-L1(+) and CD8(+) Immune Cells in HPV(+) Oropharyngeal Squamous Cell Carcinoma. Cancer Immunol. Res..

[65] Prince M.E., Sivanandan R., Kaczorowski A., Wolf G.T., Kaplan M.J., Dalerba P., Weissman I.L., Clarke M.F., Ailles L.E. (2007). Identification of a subpopulation of cells with cancer stem cell properties in head and neck squamous cell carcinoma. Proc. Natl. Acad. Sci. USA.

[66] Näsman A., Nordfors C., Grün N., Munck-Wikland E., Ramqvist T., Marklund L., Lindquist D., Dalianis T. (2013). Absent/weak CD44 intensity and positive human papillomavirus (HPV) status in oropharyngeal squamous cell carcinoma indicates a very high survival. Cancer Med..

[67] Wang Y., Springer S., Mulvey C.L., Silliman N., Schaefer J., Sausen M., James N., Rettig E.M., Guo T., Pickering C.R. (2015). Detection of somatic mutations and HPV in the saliva and plasma of patients with head and neck squamous cell carcinomas. Sci. Transl. Med..

[68] Khandelwal A.R., Greer A.H., Hamiter M., Fermin J.M., McMullen T., Moore-Medlin T., Mills G., Flores J.M., Yin H., Nathan C.O. (2020). Comparing cell-free circulating tumor DNA mutational profiles of disease-free and nonresponders patients with oropharyngeal squamous cell carcinoma. Laryngoscope Investig. Otolaryngol..

[69] Aye L., Bryan M.E., Das D., Hirayama S., Al-Inaya Y., Mendel J., Naegele S., Efthymiou V., Alzumaili B., Faquin W.C. (2024). Multi-feature next-generation liquid biopsy for diagnosis and prognosis in HPV-associated head and neck cancer. J. Clin. Oncol..

[70] Poellmann M.J., Bu J., Kim D., Iida M., Hong H., Wang A.Z., Wheeler D.L., Kimple R.J., Hong S. (2023). Circulating tumor cell abundance in head and neck squamous cell carcinoma decreases with successful chemoradiation and cetuximab treatment. Cancer Lett..

[71] Koenigs M.B., Lefranc-Torres A., Bonilla-Velez J., Patel K.B., Hayes D.N., Glomski K., Busse P.M., Chan A.W., Clark J.R., Deschler D.G. (2019). Association of estrogen receptor alpha expression with survival in oropharyngeal cancer following chemoradiation therapy. J. Natl. Cancer Inst..

[72] Kano M., Kondo S., Wakisaka N., Wakae K., Aga M., Moriyama-Kita M., Ishikawa K., Ueno T., Nakanishi Y., Hatano M. (2019). Expression of estrogen receptor alpha is associated with pathogenesis and prognosis of human papillomavirus-positive oropharyngeal cancer. Int. J. Cancer..

[73] Kwon S., Ahn S.H., Jeong W.J., Jung Y.H., Bae Y.J., Paik J.H., Chung J.H., Kim H. (2020). Estrogen receptor α as a predictive biomarker for survival in human papillomavirus-positive oropharyngeal squamous cell carcinoma. J. Transl. Med..

[74] Beaty B.T., Moon D.H., Shen C.J., Amdur R.J., Weiss J., Grilley-Olson J., Patel S., Zanation A., Hackman T.G., Thorp B. (2020). PIK3CA Mutation in HPV-Associated OPSCC Patients Receiving Deintensified Chemoradiation. JNCI J. Natl. Cancer Inst..

[75] Chiosea S.I., Grandis J.R., Lui V.W.Y., Diergaarde B., Maxwell J.H., Ferris R.L., Kim S.W., Luvison A., Miller M., Nikiforova M.N. (2013). PIK3CA, HRAS and PTEN in human papillomavirus positive oropharyngeal squamous cell carcinoma. BMC Cancer.

[76] Hanna G.J., Supplee J.G., Kuang Y., Mahmood U., Lau C.J., Haddad R.I., Jänne P.A., Paweletz C. (2018). Plasma HPV cell-free DNA monitoring in advanced HPV-associated oropharyngeal cancer. Ann. Oncol..

[77] Cochicho D., Esteves S., Rito M., Silva F., Martins L., Montalvão P., Cunha M., Magalhães M., Gil da Costa R.M., Felix A. (2022). PIK3CA Gene Mutations in HNSCC: Systematic Review and Correlations with HPV Status and Patient Survival. Cancers.

[78] Sewell A., Brown B., Biktasowa A., Mills G.B., Lu Y., Tyson D.R., Issaeva N., Yarbrough W.G. (2014). Reverse-phase protein array profiling of oropharyngeal cancer and significance of PIK3CA mutations in HPV-associated head and neck cancer. Clin. Cancer Res..

[79] Westra W.H., Taube J.M., Poeta M.L., Begum S., Sidransky D., Koch W.M. (2008). Inverse relationship between human papillomavirus-16 infection and disruptive p53 gene mutations in squamous cell carcinoma of the head and neck. Clin. Cancer Res..

[80] Boscolo-Rizzo P., Schroeder L., Sacchetto V., Holzinger D., Da Mosto M.C., Tirelli G., Dal Cin E., Mantovani M., Menegaldo A., Del Mistro A. (2019). Absence of disruptive TP53 mutations in high-risk human papillomavirus-driven neck squamous cell carcinoma of unknown primary. Head Neck.

[81] Mirghani H., Amen F., Moreau F., Guigay J., Hartl D.M., Lacau St Guily J. (2014). Oropharyngeal cancers: Relationship between epidermal growth factor receptor alterations and human papillomavirus status. Eur. J. Cancer.

[82] Hong A., Dobbins T., Lee C.S., Jackson E., Clark J., Armstrong B., Harnett G., Milross C., O’Brien C., Rose B. (2010). Relationships between epidermal growth factor receptor expression and human papillomavirus status as markers of prognosis in oropharyngeal cancer. Eur. J. Cancer..

[83] Vainshtein J.M., Spector M.E., McHugh J.B., Wong K.K., Walline H.M., Byrd S.A., Komarck C.M., Ibrahim M., Eisbruch A., Stenmark M.H. (2014). Refining risk stratification for locoregional failure after chemoradiotherapy in human papillomavirus-associated oropharyngeal cancer. Oral Oncol..

[84] Young R.J., Rischin D., Fisher R., McArthur G.A., Fox S.B., Peters L.J., Corry J., Lim A., Waldeck K., Solomon B. (2011). Relationship between epidermal growth factor receptor status, p16(INK4A), and outcome in head and neck squamous cell carcinoma. J. Clin. Oncol..

[85] Liu X., Liu P., Chernock R.D., Yang Z., Lang Kuhs K.A., Lewis J.S., Luo J., Li H., Gay H.A., Thorstad W.L. (2021). A MicroRNA Expression Signature as Prognostic Marker for Oropharyngeal Squamous Cell Carcinoma. J. Natl. Cancer Inst..

[86] Hui A.B., Lin A., Xu W., Waldron L., Perez-Ordonez B., Weinreb I., Shi W., Bruce J., Huang S.H., O’Sullivan B. (2013). Potentially prognostic miRNAs in HPV-associated oropharyngeal carcinoma. Clin. Cancer Res..

[87] Liu W., Zhang K., Wei P., Hu Y., Peng Y., Fang X., He G., Wu L., Chao M., Wang J. (2018). Correlation between miR-200 Family Overexpression and Cancer Prognosis. J. Dis. Markers.

[88] Nulton T.J., Kim K.K., DiNardo L.J., Morgan I.M., Windle B. (2018). Patients with integrated HPV16 in head and neck cancer show poor survival. Oral Oncol..

[89] Walline H.M., Komarck C.M., McHugh J.B., Bellile E.L., Brenner J.C., Prince M.E., McKean E.L., Chepeha D.B., Wolf G.T., Worden F.P. (2016). Genomic Integration of High-Risk HPV Alters Gene Expression in Oropharyngeal Squamous Cell Carcinoma. Mol. Cancer Res..

[90] Koneva L.A., Zhang Y., Virani S., Hall P.B., McHugh J.B., Chepeha D.B., Wolf G.T., Carey T.E., Rozek L.S., Sartor M. (2018). HPV Integration in HNSCC Correlates with Survival Outcomes, Immune Response Signatures, and Candidate Drivers. Mol. Cancer Res..

[91] Mainguené J., Vacher S., Kamal M., Hamza A., Masliah-Planchon J., Baulande S., Ibadioune S., Borcoman E., Cacheux W., Calugaru V. (2022). Human papilloma virus integration sites and genomic signatures in head and neck squamous cell carcinoma. Mol. Oncol..

[92] Stevenson A., Wakeham K., Pan J., Kavanagh K., Millan D., Bell S., McLellan D., Graham S.V., Cuschieri K. (2020). Droplet digital PCR quantification suggests that higher viral load correlates with improved survival in HPV+OPC. J. Clin. Virol..

[93] Veitía D., Liuzzi J., Ávila M., Rodriguez I., Toro F., Correnti M. (2020). Association of viral load and physical status of HPV-16 with survival of patients with head and neck cancer. Ecancermedicalscience.

[94] Villarmé A., Ebran N., Pace-Loscos T., Schiappa R., Mignot A., Bozec A., Sudaka-Bahadoran A., Saada-Bouzid E., Culié D. (2024). Prognostic impact of intra tumoral HPV-16 viral load in oropharyngeal squamous cell carcinomas. Oral Oncol..

[95] Burkitt K. (2023). Role of DNA Methylation Profiles as Potential Biomarkers and Novel Therapeutic Targets in Head and Neck Cancer. Cancers.

[96] Nakagawa T., Kurokawa M., Mima M., Imamoto S., Mizokami H., Kondo S., Okamoto Y., Misawa K., Hanazawa T., Kaneda A. (2021). DNA Methylation and HPV-Associated Head and Neck Cancer. Microorganisms.

[97] Allen C.T., Ricker J.L., Chen Z., van Waes C. (2007). Role of activated nuclear factor-kappaB in the pathogenesis and therapy of squamous cell carcinoma of the head and neck. Head Neck.

[98] Schrank T.P., Kothari A., Weir W.H., Stepp W.H., Rehmani H., Liu X., Wang X., Sewell A., Li X., Tasoulas J. (2023). Noncanonical HPV carcinogenesis drives radiosensitization of head and neck tumors. Proc. Natl. Acad. Sci. USA.

[99] Hong A.M., Dobbins T.A., Lee C.S., Jones D., Fei J., Clark J.R., Armstrong B.K., Harnett G.B., Milross C.G., Tran N. (2010). Use of cyclin D1 in conjunction with human papillomavirus status to predict outcome in oropharyngeal cancer. Int. J. Cancer.

[100] Scantlebury J.B., Luo J., Thorstad W.L., El-Mofty S.K., Lewis J.S. (2013). Cyclin D1-a prognostic marker in oropharyngeal squamous cell carcinoma that is tightly associated with high-risk human papillomavirus status. Hum. Pathol..

[101] Gross A.M., Orosco R.K., Shen J.P., Egloff A.M., Carter H., Hofree M., Choueiri M., Coffey C.S., Lippman S.M., Hayes D.N. (2014). Multi-tiered genomic analysis of head and neck cancer ties TP53 mutation to 3p loss. Nat. Genet..

[102] Rittà M., De Andrea M., Mondini M., Mazibrada J., Giordano C., Pecorari G., Garzaro M., Landolfo V., Schena M., Chiusa L. (2009). Cell cycle and viral and immunologic profiles of head and neck squamous cell carcinoma as predictable variables of tumor progression. Head Neck.

[103] Sittel C., Ruiz S., Volling P., Kvasnicka H.M., Jungehülsing M., Eckel H.E. (1999). Prognostic significance of Ki-67, PCNA and p53 in cancer of the oropharynx and oral cavity. Oral Oncol..

[104] Maebayashi T., Ishibashi N., Aizawa T., Sakaguchi M., Saito T., Kawamori J., Tanaka Y., Hirotani Y., Homma T. (2019). Roles of Ki-67 and p16 as biomarkers for unknown primary head and neck squamous cell carcinoma. Eur. Arch. Otorhinolaryngol..

[105] Ho A.S., Robinson A., Shon W., Laury A., Raedschelders K., Venkatraman V., Holewinski R., Zhang Y., Shiao S.L., Chen M.M. (2022). Comparative Proteomic Analysis of HPV(+) Oropharyngeal Squamous Cell Carcinoma Recurrences. J. Proteome Res..

[106] Shea B.J., Grimshaw J.M., Wells G.A., Boers M., Andersson N., Hamel C., Porter A.C., Tugwell P., Moher D., Bouter L.M. (2007). Development of AMSTAR: A measurement tool to assess the methodological quality of systematic reviews. BMC Med. Res. Methodol..

[107] Mehanna H., Taberna M., von Buchwald C., Tous S., Brooks J., Mena M., Morey F., Grønhøj C., Rasmussen J.H., Garset-Zamani M. (2023). Prognostic implications of p16 and HPV discordance in oropharyngeal cancer (HNCIG-EPIC-OPC): A multicentre, multinational, individual patient data analysis. Lancet Oncol..

[108] Bishop J.A., Ma X.-J., Wang H., Luo Y., Illei P.B., Begum S., Taube J.M., Koch W.M., Westra W.H. (2012). Detection of transcriptionally active high-risk HPV in patients with head and neck squamous cell carcinoma as visualized by a novel E6/E7 mRNA in situ hybridization method. Am. J. Surg. Pathol..

[109] Worden F.P., Kumar B., Lee J.S., Wolf G.T., Cordell K.G., Taylor J.M., Urba S.G., Eisbruch A., Teknos T.N., Chepeha D.B. (2008). Chemoselection as a strategy for organ preservation in advanced oropharynx cancer: Response and survival positively associated with HPV16 copy number. J. Clin. Oncol..

[110] Posner M.R., Botzler J., Takahashi M. (2023). Quarterback trials: A phase 2 series of sequential studies of induction chemotherapy followed by reduced dose chemoradiation for human papillomavirus positive oropharynx cancer. J. Clin. Oncol..

[111] Lacas B., Carmel A., Landais C., Wong S.J., Licitra L., Tobias J.S., Burtness B., Ghi M.G., Cohen E.E.W., Grau C. (2021). Meta-analysis of chemotherapy in head and neck cancer (MACH-NC): An update on 107 randomized trials and 19,805 patients, on behalf of MACH-NC Group. Radiother. Oncol..

[112] Brizel D.M., Dodge R.K., Clough R.W., Dewhirst M.W. (1999). Oxygenation of head and neck cancer: Changes during radiotherapy and impact on treatment outcome. Radiother. Oncol..

[113] Löck S., Perrin R., Seidlitz A., Bandurska-Luque A., Zschaeck S., Zöphel K., Krause M., Steinbach J., Kotzerke J., Zips D. (2017). Residual tumour hypoxia in head-and-neck cancer patients undergoing primary radiochemotherapy, final results of a prospective trial on repeat FMISO-PET imaging. Radiother. Oncol..

[114] Nicolay N.H., Wiedenmann N., Mix M., Weber W.A., Werner M., Grosu A.L., Kayser G. (2020). Correlative analyses between tissue-based hypoxia biomarkers and hypoxia PET imaging in head and neck cancer patients during radiochemotherapy—Results from a prospective trial. Eur. J. Nucl. Med. Mol. Imaging.

[115] Zips D., Zöphel K., Abolmaali N., Perrin R., Abramyuk A., Haase R., Appold S., Steinbach J., Kotzerke J., Baumann M. (2012). Exploratory prospective trial of hypoxia-specific PET imaging during radiochemotherapy inpatients with locally advanced head-and-neck cancer. Radiother. Oncol..

[116] Swartz J.E., Pothen A.J., van Kempen P.M., Stegeman I., Formsma F.K., Cann E.M., Willems S.M., Grolman W. (2016). Poor prognosis in human papillomavirus-positive oropharyngeal squamous cell carcinomas that overexpress hypoxia inducible factor-1α. Head Neck.

[117] Zschaeck S., Löck S., Hofheinz F., Zips D., Saksø Mortensen L., Zöphel K., Troost E.G.C., Boeke S., Saksø M., Mönnich D. (2020). Individual patient data meta-analysis of FMISO and FAZA hypoxia PET scans from head and neck cancer patients undergoing definitive radio-chemotherapy. Radiother. Oncol..

[118] Kendi A.T., Brandon D., Switchenko J., Wadsworth J.T., El-Deiry M.W., Saba N.F., Schuster D.M., Subramaniam R.M. (2017). Head and neck PET/CT therapy response interpretation criteria (Hopkins criteria)-external validation study. Am. J. Nucl. Med. Mol. Imaging.

[119] Chotchutipan T., Rosen B.S., Hawkins G., Lee J.Y., Saripalli A.L., Thakkar D., Eisbruch A., El Naqa I., Mierzwa M.L. (2019). Volumetric ^18^F-FDG-PET parameters predict locoregional failure in low-risk HPV-related oropharyngeal cancer patients following definitive chemoradiation therapy. Head Neck.

[120] Rosen B.S., Wilkie J.R., Sun Y., Ibrahim M., Casper K.A., Miller J.E., Chotchutipan T., Stucken C.L., Bradford C., Prince M.E.P. (2021). CT and FDG-PET radiologic biomarkers in p16+ oropharyngeal squamous cell carcinoma patients treated with definitive chemoradiotherapy. Radiother. Oncol..

[121] Gouw Z.A.R., La Fontaine M.D., van Kranen S., van de Kamer J.B., Vogel W.V., van Werkhoven E., Sonke J.J., Al-Mamgani A. (2019). The Prognostic Value of Baseline 18F-FDG PET/CT in Human Papillomavirus–Positive Versus Human Papillomavirus–Negative Patients with Oropharyngeal Cancer. Clin. Nucl. Med..

[122] Moan J.M., Amdal C.D., Malinen E., Svestad J.G., Bogsrud T.V., Dale E. (2019). The prognostic role of 18F-fluorodeoxyglucose PET in head and neck cancer depends on HPV status. Radiother. Oncol..

[123] Castaldi P., Rufini V., Bussu F., Miccichè F., Dinapoli N., Autorino R., Lago M., De Corso E., Almadori G., Galli J. (2012). Can “early” and “late” 18F-FDG PET-CT be used as prognostic factors for the clinical outcome of patients with locally advanced head and neck cancer treated with radio-chemotherapy?. Radiother. Oncol..

[124] Martens R.M., Koopman T., Lavini C., Brug T.V., Zwezerijnen G.J.C., Marcus J.T., Vergeer M.R., Leemans C.R., Bree R., Graaf P. (2022). Early response prediction of multiparametric functional MRI and18F-FDG-PET in patients with headand neck squamous cell carcinoma treated with (chemo)radiation. Cancers.

[125] Dirix P., Vandecaveye V., Keyzer F.D., Stroobants S., Hermans R., Nuyts S. (2009). Dose painting in radiotherapyfor head and neck squamous cell carcinoma: Value of repeated functionalimaging with (18)F-FDG PET, (18)F-fluoromisonidazole PET, diffusion-weighted MRI, and dynamic contrast-enhanced MRI. J. Nucl. Med..

[126] Pak K., Cheon G.J., Nam H.Y., Kim S.J., Kang K.W., Chung J.K. (2014). Prognostic value of metabolic tumor volume and total lesion glycolysis in head and neck cancer: A systematic review and meta-analysis. J. Nucl. Med..

[127] Mowery Y.M., Vergalasova I., Rushing C.N., Choudhury K.R., Niedzwiecki D., Wu Q., Yoo D.S., Das S., Wong T.Z., Brizel D.M. (2020). Early18F-FDG-PET re-sponse during radiation therapy for HPV-related oropharyngeal cancer maypredict disease recurrence. Int. J. Radiat. Oncol. Biol. Phys..

[128] Mena E., Taghipour M., Sheikhbahaei S., Jha A.K., Rahmim A., Solnes L. (2017). Value of Intratumoral Metabolic Heterogeneity and Quantitative 18F-FDG PET/CT Parameters to Predict Prognosis in Patients with HPV-Positive Primary Oropharyngeal Squamous Cell Carcinoma. Clin. Nucl. Med..

[129] Lim R., Eaton A., Lee N.Y., Setton J., Ohri N., Rao S., Wong R., Fury M., Scoder H. (2012). 18F-FDG PET/CT metabolic tumor volume and total lesion glycolysis predict outcome in oropharyngeal squamous cell carcinoma. J. Nucl. Med..

[130] Pollom E.L., Song J., Durkee B.Y., Aggarwal S., Bui T., von Eyben R., Aggarwal S., Bui T., von Eyben R., Li R. (2016). Prognostic value of midtreatment FDG-PET in oropharyngeal cancer. Head Neck.

[131] Leung E., Han K., Zou J., Zhao Z., Zheng Y., Wang T.T., Rostami A., Siu L.L., Pugh T.J., Bratman S.V. (2021). HPV Sequencing Facilitates Ultrasensitive Detection of HPV Circulating Tumor DNA. Clin. Cancer Res..

[132] Jeannot E., Becette V., Campitelli M., Calméjane M.A., Lappartient E., Ruff E., Saada S., Holmes A., Bellet D., Sastre-Garau X. (2016). Circulating human papillomavirus DNA detected using droplet digital PCR in the serum of patients diagnosed with early stage human papillomavirus-associated invasive carcinoma. J. Pathol. Clin. Res..

[133] Hanna G.J., Kacew A., Chau N.G., Shivdasani P., Lorch J.H., Uppaluri R., Haddad R.I., MacConaill L.E. (2018). Improved outcomes in PI3K-pathwayaltered metastatic HPV oropharyngeal cancer. JCI Insight.

[134] Haring C.T., Brummel C., Bhambhani C., Jewell B., Neal M.H., Bhangale A., Casper K., Malloy K., McLean S., Shuman A. (2021). Implementation of human papillomavirus circulating tumor DNA to identify recurrence during treatment de-escalation. Oral Oncol..

[135] Cao H., Banh A., Kwok S., Shi X., Wu S., Krakow T., Khong B., Bavan B., Bala R., Pinsky B.A. (2012). Quantitation of Human Papillomavirus DNA in Plasma of Oropharyngeal Carcinoma Patients. Int. J. Radiat. Oncol. Biol. Phys..

[136] Rettig E.M., Wang A.A., Tran N.-A., Carey E., Dey T., Schoenfeld J.D., Sehgal K., Guenette J.P., Margalit D.N., Sethi R. (2022). Association of Pretreatment Circulating Tumor Tissue–Modified Viral HPV DNA with Clinicopathologic Factors in HPV-Positive Oropharyngeal Cancer. JAMA Otolaryngol.–Head Neck Surg..

[137] Naegele S., Ruiz-Torres D.A., Zhao Y., Goss D., Faden D.L. (2023). Comparing the Diagnostic Performance of Quantitative PCR, Digital Droplet PCR, and Next-Generation Sequencing Liquid Biopsies for Human Papillomavirus-Associated Cancers. J. Mol. Diagn..

[138] Allevato M.M., Smith J.D., Brenner M.J., Chinn S.B. (2023). Tumor-Derived Exosomes and the Role of Liquid Biopsy in Human Papillomavirus Oropharyngeal Squamous Cell Carcinoma. Cancer J..

[139] Galiveti C.R., Kuhnell D., Biesiada J., Zhang X., Kelsey K.T., Takiar V., Tang A.L., Wise-Draper T.M., Medvedovic M., Kasper S. (2023). Small Extravesicular MicroRNA in Head and Neck Squamous Cell Carcinoma and Its Potential as a Liquid Biopsy for Early Detection. Head Neck.

[140] Pellini B., Szymanski J., Chin R. (2020). Liquid biopsies using circulating tumor DNA in NSCLC. Thorac. Surg. Clin..

[141] Kann B.H., Likitlersuang J., Bontempi D., Ye Z., Aneja S., Bakst R., Kelly H.R., Juliano A.F., Payabvash S., Guenette J.P. (2023). Screening for extranodal extension in HPV-associated oropharyngeal carcinoma: Evaluation of a CT-based deep learning algorithm in patient data from a multicentre, randomised de-escalation trial. Lancet Digit Health.

[142] Henson C., Abou-Foul E.Y., lastonbury C., Huang S.H., King A.D., Lydiatt W.M., McDowell L., Nagelschneider A.A., Nankivell P.C., O’Sullivan B. (2024). Criteria for the diagnosis of extranodal extension detected on radiological imaging in head and neck cancer: HNCIG international consensus recommendations. Lancet Oncol..

[143] Glogauer J., Kohanzadeh A., Feit A., Fournier J.E., Zians A., Somogyi D.Z. (2023). The Use of Radiomic Features to Predict Human Papillomavirus (HPV) Status in Head and Neck Tumors: A Review. Cureus.

[144] Mayerhoefer M.E., Materka A., Langs G., Häggström I., Szczypiński P., Gibbs P., Cook G. (2020). Introduction to Radiomics. J. Nucl. Med..

[145] Lambin P., Leijenaar R.T.H., Deist T.M., Peerlings J., de Jong E.E.C., van Timmeren J., Sanduleanu S., Larue R.T.H.M., Even A.J.G., Jochems A. (2017). Radiomics: The bridge between medical imaging and personalized medicine. Nat. Rev. Clin. Oncol..

[146] Bagher-Ebadian H., Siddiqui H., Ghanem H., Ghanem A.I., Zhu S., Lu M., Movsas B., Chetty I.J. (2022). Radiomics outperforms clinical factors in characterizing human papilloma virus (HPV) for patients with oropharyngeal squamous cell carcinomas. Biomed. Phys. Eng. Express.

[147] Aerts H.J., Velazquez E.R., Leijenaar R.T., Parmar C., Grossmann P., Carvalho S., Bussink J., Monshouwer R., Haibe-Kains B., Rietveld D. (2014). Decoding tumour phenotype by noninvasive imaging using a quantitative radiomics approach. Nat. Commun..

[148] Wang P., Wang X., Zhang M., Li G., Zhao N., Qiao Q. (2022). Combining the radiomics signature and HPV status for the risk strateification of patients with OPC. Oral Dis..

[149] Kwan J.Y.Y., Su J., Huang S.H., Ghoraie L.S., Ghoraie L.S., Xu W., Chan B., Yip K.W., Giuliani M., Bayley A. (2018). Radiomic Biomarkers to Refine Risk Models for Distant Metastasis in HPV-related Oropharyngeal Carcinoma. Int. J. Radiat. Oncol. Biol. Phys..

[150] Volpe S., Gaeta A., Colombo F., Zaffaroni M., Mastroleo F., Vincini M.G., Pepa M., Isaksson L.J., Turturici I., Marvaso G. (2023). Blood- and Imaging-Derived Biomarkers for Oncological Outcome Modelling in Oropharyngeal Cancer: Exploring the Low-Hanging Fruit. Cancers.

[151] Haider S.P., Zeevi T., Baumeister P., Reichel C., Sharaf K., Forghani R., Kann B.H., Judson B.L., Prasad M.L., Burtness B. (2020). Potential Added Value of PET/CT Radiomics for Survival Prognostication beyond AJCC 8th Edition Staging in Oropharyngeal Squamous Cell Carcinoma. Cancers.

[152] Mori M., Deantoni C., Olivieri M., Spezi E., Chiara A., Baroni S., Picchio M., Del Vecchio A., Di Muzio N.G., Fiorino C. (2023). External validation of an ^18^F-FDG-PET radiomic model predicting survival after radiotherapy for oropharyngeal cancer. Eur. J. Nucl. Med. Mol. Imaging.

[153] Bologna M., Corino V., Cavalieri S., Calareso G., Gazzani S.E., Poli T., Ravanelli M., Mattavelli D., de Graaf P., Nauta I. (2023). Prognostic radiomic signature for head and neck cancer: Development and validation on a multi-centric MRI dataset. Radiother. Oncol..

[154] Ger R.B., Zhou S., Elgohari B., Elhalawani H., Mackin D.M., Meier J.G., Nguyen C.M., Anderson B.M., Gay C., Ning J. (2019). Radiomics features of the primary tumor fail to improve prediction of overall survival in large cohorts of CT- and PET-imaged head and neck cancer patients. PLoS ONE.

[155] Lewis J.S., Scantlebury J.B., Luo J., Thorstad W.L. (2012). Tumor cell anaplasia and multinucleation are predictors of disease recurrence in oropharyngeal squamous cell carcinoma, including among just the human papillomavirus-related cancers. Am. J. Surg. Pathol..

[156] Koyuncu C.F., Lu C., Bera K., Zhang Z., Xu J., Toro P., Corredor G., Chute D., Fu P., Thorstad W.L. (2021). Computerized tumor multinucleation index (MuNI) is prognostic in p16+ oropharyngeal carcinoma: A multi-site validation study. J. Clin. Investig..

[157] Molony P., Werner R., Martin C., Callanan D., Sheahan P., Heffron C., Feeley L. (2020). Tumour cell anaplasia and multinucleation as prognosticators in oropharyngeal squamous cell carcinoma. Head Neck Pathol..

[158] Corredor G., Toro P., Koyuncu C., Lu C., Buzzy C., Bera K., Fu P., Mehrad M., Ely K.A., Mokhtari M. (2022). An imaging biomarker of tumor-infiltrating lymphocytes to risk-stratify patients with HPV-associated oropharyngeal cancer. J. Natl. Cancer Inst..

[159] Hue J., Valinciute Z., Thavaraj S., Veschini L. (2023). Multifactorial estimation of clinical outcome in HPV-associated oropharyngeal squamous cell carcinoma via automated image analysis of routine diagnostic H&E slides and neural network modelling. Oral Oncol..

[160] Charles K.A., Harris B.D.W., Haddad C.R., Clarke S.J., Guminski A., Stevens M., Dodds T., Gill A.J., Back M., Veivers D. (2016). Systemic inflammation is an independent predictive marker of clinical outcomes in mucosal squamous cell carcinoma of the head and neck in oropharyngeal and non-oropharyngeal patients. BMC Cancer.

[161] Ma S.J., Yu H., Khan M., Santhosh S., Chatterjee U., Iovoli A., Farrugia M., Mohammadpour H., Wooten K., Gupta V. (2022). Evaluation of Optimal Threshold of Neutrophil-Lymphocyte Ratio and Its Association with Survival Outcomes Among Patients with Head and Neck Cancer. JAMA Netw. Open.

[162] Ward M.J., Thirdborough S.M., Mellows T., Riley C., Harris S., Suchak K., Webb A., Hampton C., Patel N.N., Randall C.J. (2014). Tumour-infiltrating lymphocytes predict for outcome in HPV-positive oropharyngeal cancer. Br. J. Cancer..

[163] Yin L.X., Rivera M., Garcia J.J., Bartemes K.R., Lewis D.B., Lohse C.M., Routman D.M., Ma D.J., Moore E.J., Van Abel K.M. (2023). Impact of Tumor-Infiltrating Lymphocytes on Disease Progression in Human Papillomavirus-Related Oropharyngeal Carcinoma. Otolaryngol. Head Neck Surg..

[164] Oguejiofor K., Hall J., Slater C., Betts G., Hall G., Slevin N., Dovedi S., Stern P.L., West C.M. (2015). Stromal infiltration of CD8 T cells is associated with improved clinical outcome in HPV-positive oropharyngeal squamous carcinoma. Br. J. Cancer..

[165] Zeng Y.F., Barret J.W., Shammas-Toma M., Shammas-Toma M., De Cecco L., Serafini M.S., Cavalieri S., Licitra L., Hoebers F., Brakenhoff R.H. (2022). Immune-based classification of HPV-associated oropharyngeal cancer with implications for biomarker-driven treatment de-intensification. eBioMedicine.

[166] Smith J.D., Ludwig M.L., Bhangale A.D., Brummel C., Swiecicki P.L., Worden F.P., Chinn S.B., Stucken C.L., Rosko A.J., Prince M.E.P. (2023). Tumor immune microenvironment alterations using induction cetuximab in a phase II trial of deintensified therapy for p16-positive oropharynx cancer. Head Neck.

[167] Jie H.B., Srivastava R.M., Argiris A., Bauman J.E., Kane L.P., Ferris R.L. (2017). Increased PD-1+ and TIM-3+ TILs during Cetuximab Therapy Inversely Correlate with Response in Head and Neck Cancer Patients. Cancer Immunol. Res..

[168] Li S., Garb B.F., Qin T., Soppe S.E., Lopez E., Patil S., D’Silva N.J., Rozek L.S., Sartor M.A. (2024). Tumor Subtype Classification Tool for HPV-associated. Head and Neck Cancers. bioRxiv.

[169] Locati L.D., Serafini M.S., Iannò M.F., Carenzo A., Orlandi E., Resteghin C., Cavalieri S., Bossi P., Canevari S., Licitra L. (2019). Mining of Self-Organizing Map Gene-Expression Portraits Reveals Prognostic Stratification of HPV-Positive Head and Neck Squamous Cell Carcinoma. Cancers.

[170] Pim D., Massimi P., Banks L. (1997). Alternatively spliced HPV-18 E6* protein inhibits E6 mediated degradation of p53 and suppresses transformed cell growth. Oncogene.

[171] Maharajan N., Benyamien-Roufaeil D.S., Brown R.A., Brown R.A., Portney B.A., Banerjee A., Zalzman M. (2025). Cancer stem cell mechanisms and targeted therapeutic strategies in head and neck squamous cell carcinoma. Cancer Lett..

[172] Rietbergen M.M., Martens-de Kemp S.R., Bloemena E., Baatenburg de Jong R.J., Leemans C.R., Braakhuis B.J., Brakenhoff R.H. (2014). Cancer stem cell enrichment marker CD98: A prognostic factor for survival in patients with human papillomavirus-positive oropharyngeal cancer. Eur. J. Cancer..

[173] Kim S.I., Woo S.R., Lee M.K., Lee Y.C., Lee J.W., Kong M., Ko S.G., Eun Y.G. (2022). Association between cancer stem cell gene expression signatures and prognosis in head and neck squamous cell carcinoma. BMC Cancer.

[174] Poeta M.L., Manola J., Goldwasser M.A., Forastiere A., Benoit N., Califano J.A., Ridge J.A., Goodwin J., Kenady D., Saunders J. (2007). TP53 Mutations and Survival in Squamous-Cell Carcinoma of the Head and Neck. N. Engl. J. Med..

[175] Nathan C.A., Khandelwal A.R., Wolf G.T., Rodrigo J.P., Mäkitie A.A., Saba N.F., Forastiere A.A., Bradford C.R., Ferlito A. (2022). TP53 mutations in head and neck cancer. Mol. Carcinog..

[176] Kumar B., Cordell K.G., Lee J.S., Worden F.P., Prince M.E., Tran H.H., Wolf G.T., Urba S.G., Chepeha D.B., Teknos T.N. (2008). EGFR, p16, HPV titer, Bcl-xL and p53, sex, and smoking as indicators of response to therapy and survival in oropharyngeal cancer. J. Clin. Oncol..

[177] Kong C.S., Narasimhan B., Cao H., Kwok S., Erickson J.P., Koong A., Pourmand N., Le Q.T. (2009). The relationship between human papillomavirus status and other molecular prognostic markers in head and neck squamous cell carcinomas. Int. J. Radiat. Oncol. Biol. Phys..

[178] Perisanidis C. (2017). Prevalence of EGFR Tyrosine Kinase Domain Mutations in Head and Neck Squamous Cell Carcinoma: Cohort Study and Systematic Review. In Vivo.

[179] Vojtechova Z., Sabol I., Salakova M., Turek L., Grega M., Smahelova J., Vencalek O., Lukesova E., Klozar J., Tachezy R. (2016). Analysis of the integration of human papillomaviruses in head and neck tumours in relation to patients’ prognosis. Int. J. Cancer.

[180] Lim M.Y., Dahlstrom K.R., Sturgis E.M., Li G. (2016). Human papillomavirus integration pattern and demographic, clinical, and survival characteristics of patients with oropharyngeal squamous cell carcinoma. Head Neck.

[181] Yarbrough W.G., Schrank T.P., Burtness B.A., Issaeva N. (2024). De-Escalated Therapy and Early Treatment of Recurrences in HPV-Associated Head and Neck Cancer: The Potential for Biomarkers to Revolutionize Personalized Therapy. Viruses.

[182] Fan J., Fu Y., Peng W., Shen Y., Guo E., Lu F., Zhou S., Liu S., Yang B., Qin X. (2023). Multi-omics characterization of silent and productive HPV integration in cervical cancer. Cell Genom..

[183] Rasmussen K.D., Helin K. (2016). Role of TET enzymes in DNA methylation, development, and cancer. Genes Dev..

[184] Misawa K., Imai A., Mochizuki D., Mima M., Endo S., Misawa Y., Kanazawa T., Mineta H. (2018). Association of TET3 epigenetic inactivation with head and neck cancer. Oncotarget.

[185] Ramachandran B. (2017). Functional association of oestrogen receptors with HPV infection in cervical carcinogenesis. Endocr. Relat. Cancer.

[186] Jie W., Bai J., Yan J., Chi Y., Li B.B. (2021). Multi-Site Tumour Sampling Improves the Detection of Intra-Tumour Heterogeneity in Oral and Oropharyngeal Squamous Cell Carcinoma. Front. Med..

[187] Morris L.G.T., Chandramohan R., West L., Zehir A., Chakravarty D., Pfister D.G., Wong R.J., Lee N.Y., Sherman E.J., Baxi S.S. (2017). The Molecular Landscape of Recurrent and Metastatic Head and Neck Cancers: Insights From a Precision Oncology Sequencing Platform. JAMA Oncol..

[188] Kim H.A.J., Shaikh M.H., Lee M., Zeng P.Y.F., Sorgini A., Akintola T., Deng X., Jarycki L., Khan H., MacNeil D. (2021). 3p Arm Loss and Survival in Head and Neck Cancer: An Analysis of TCGA Dataset. Cancers.

[189] Yin Y., Song W.W., Wang Y., Zhao W., Wu J., Xu W. (2018). MicroRNA-200 families and prognostic value in various carcinomas: A systematic review and meta-analysis. Aging Med..

[190] Prusinkiewicz M.A., Gameiro S.F., Ghasemi F., Dodge M.J., Zeng P.Y.F., Maekebay H., Barrett J.W., Nichols A.C., Mymryk J.S. (2020). Survival-Associated Metabolic Genes in Human Papillomavirus-Positive Head and Neck Cancers. Cancers.

[191] Yap T.A., Gerlinger M., Futreal P.A., Pusztai L., Swanton C. (2012). Intratumor heterogeneity: Seeing the wood for the trees. Sci. Transl. Med..

[192] Mehanna H., Rapozo D., von Zeidler S.V., Harrington K.J., Winter S.C., Hartley A., Nankivell P., Schache A.G., Sloan P., Odell E.W. (2023). Developing and validating a multivariable prognostic-predictive classifier for treatment escalation of oropharyngeal squamous cell carcinoma: The PREDICTR-OPC study. Clin. Cancer Res..

[193] Makitie A.A., Alabi R.O., Ng S.P., Takes R.P., Robbins K.T., Ronen O., Shaha A.R., Bradley P.J., Saba N.F., Nuyts S. (2023). Artificial intelligence in head and neck cancer: A systematic review of systematic reviews. Adv. Ther..

[194] Diao P., Dai Y., Wang A., Bu X., Wang Z., Li J., Wu Y., Jiang H., Wang Y., Cheng J. (2024). Integrative multiomics analyses identify molecular subtypes of head and neck squamous cell carcinoma with distinct therapeutic vulnerabilities. Cancer Res..

[195] Madabhushi A., Lee G. (2016). Image analysis and machine learning in digital pathology: Challenges and opportunities. Med. Image Anal..

[196] Hieromnimon H.M., Trzcinska A., Wen F.T., Howard F.M., Dolezal J.M., Dyer E., Kochanny S., Schulte J.J., Wang C., Chen H. (2025). Analysis of AI foundation model features decodes the histopathologic landscape of HPV-positive head and neck squamous cell carcinomas. Oral Oncol..

[197] Song B., Leroy A., Yang K., Dam T., Wang X., Maurya H., Pathak T., Lee J., Stock S., Li X.T. (2025). Deep learning informed multimodal fusion of radiology and pathology to predict outcomes in HPV-associated oropharyngeal squamous cell carcinoma. eBioMedicine.

[198] Sher D.J., Adelstein D.J., Bajaj G.K., Brizel D.M., Cohen E.E.W., Halthore A., Harrison L.B., Lu C., Moeller B.J., Quon H. (2017). Radiation therapy for oropharyngeal squamous cell carcinoma: Executive summary of an ASTRO evidence-based clinical practice guideline. Pract. Radiat. Oncol..

[199] Feng F.Y., Kim H.M., Lyden T.H., Haxer M.J., Worden F.P., Feng M., Moyer J.S., Prince M.E., Carey T., Wolf G.T. (2010). Intensity-modulated chemoradiotherapy aiming to reduce dysphagia in patients with oropharyngeal cancer: Clinical and functional results. J. Clin. Oncol..

